# Molecular Evolution and Genetic Variation of *G2-Like* Transcription Factor Genes in Maize

**DOI:** 10.1371/journal.pone.0161763

**Published:** 2016-08-25

**Authors:** Fang Liu, Yunjian Xu, Guomin Han, Lingyan Zhou, Asif Ali, Suwen Zhu, Xiaoyu Li

**Affiliations:** Key Laboratory of Crop Biology of Anhui Province, Anhui Agricultural University, Hefei, China; Agriculture and Agri-Food Canada, CANADA

## Abstract

The productivity of maize (*Zea mays* L.) depends on the development of chloroplasts, and *G2-like* transcription factors play a central role in regulating chloroplast development. In this study, we identified 59 *G2-like* genes in the B73 maize genome and systematically analyzed these genes at the molecular and evolutionary levels. Based on gene structure character, motif compositions and phylogenetic analysis, maize *G2-like* genes (*ZmG1- ZmG59*) were divided into seven groups (I-VII). By synteny analysis, 18 collinear gene pairs and strongly conserved microsyntny among regions hosting *G2-like* genes across maize and sorghum were found. Here, we showed that the vast majority of *ZmG* gene duplications resulted from whole genome duplication events rather than tandem duplications. After gene duplication events, some *ZmG* genes were silenced. The functions of *G2-like* genes were multifarious and most genes that are expressed in green tissues may relate to maize photosynthesis. The qRT-PCR showed that the expression of these genes was sensitive to low temperature and drought. Furthermore, we analyzed differences of *ZmG*s specific to cultivars in temperate and tropical regions at the population level. Interestingly, the single nucleotide polymorphism (SNP) analysis revealed that nucleotide polymorphism associated with different temperature zones. Above all, *G2-like* genes were highly conserved during evolution, but polymorphism could be caused due to a different geographical location. Moreover, *G2-like* genes might be related to cold and drought stresses.

## Introduction

The chloroplasts of higher plants are believed to have originated from aquatic single-celled cyanobacteria more than 2.5 billion years ago [1–3]. Chloroplasts contain the green pigment chlorophyll and are responsible for the light-powered reactions of photosynthesis, upon which essentially all life depends [2–4]. Recent studies supported the view that chloroplasts were derived from a primary endosymbiotic event involving such cyanobacteria [5–8]. As a result, the regulation of chloroplast biogenesis involves cooperation between the nucleus and chloroplast. Plastids play a variety of roles in addition to functioning in photosynthesis, including roles in the synthesis of amino acids, fatty acids, purine and pyrimidine bases, terpenoids and various pigments and hormones, as well as functioning in key aspects of nitrogen and sulfur assimilation [2,9–11].

Proplastids in subepidermal meristematic cells (or etioplasts in dark-grown cotyledons) are transformed into mesophyll chloroplasts upon exposure to light [1,12]. Approximately 50% of genes are differentially expressed during this transformation [1,13–15] and transcription factors play key regulation in this process. Members of *Golden 2-like* (*G2-like*) family have been characterized with roles of regulating the formation of chloroplasts during the transition and early maturation phases [1,16]. And *G2-like* genes are indispensable for chloroplast development in angiosperms.

*G2-like* genes are members of the recently categorized GARP superfamily of transcription factors [17,18]. Within the GARP family, *G2-like* genes are monophyletic, while interestingly, gene duplications have occurred independently in monocots and eudicots [19]. Most *G2-like* genes have two domains, including a Myb-DNA binding domain (DBD; containing an HLH motif) and a C-terminal domain (containing a conserved GCT box). Several chloroplast development-related transcription factors have been reported in plants. For example, *ACCUMULATION AND REPLICATION OF CHLOROPLASTS5* encodes a cytoplasmically localized dynamin-like protein that regulates chloroplast division in *Arabidopsis thaliana* [20]. Two transposase-derived transcription factors, FAR-RED ELONGATED HYPOCOTYL3 (FHY3) and FAR-RED IMPAIRED RESPONSE1 (FAR1), are positive regulators of chlorophyll biosynthesis in *Arabidopsis* [21]. *Golden2-like (GLK)* genes regulate chloroplast development in the monocot maize (*Zea mays* L.) and in the eudicot *Arabidopsis* [4,21–24]. *GLK* genes help coregulate and synchronize the expression of a suite of nucleus photosynthetic genes and thus act to optimize photosynthetic capacity under varying environmental and developmental conditions [25]. *GLK* overexpression enhances the expression of genes related to fruit photosynthesis and chloroplast development, leading to elevated carbohydrate and carotenoid levels in ripe fruit [26]. *SlGLK2* influences photosynthesis in developing fruit, contributing to mature fruit characteristics [26]. *ZmGLK1* is thought to regulate mesophyll cell chloroplast development in C4 tissues, and *GLK* gene pairs act redundantly to promote chloroplast development in C3 species [4,23]. In addition, overexpression of *AtGLK1* (*35S*:*AtGLK1*) in *Arabidopsis* also confers resistance to the cereal pathogen *Fusarium graminearum* [27]. Maize is a model genetic system and one of the world’s highest valued crops, accounting for billions of dollars of annual agricultural revenue [28]. To further increase crop productivity, one way is to improve the stress and disease resistance. *G2-like* genes might have a function in stress and disease resistance. Although *G2-like* transcription factors were first characterized in maize [24], a systematic analysis and comparison of *G2-like* genes in maize has not previously been reported. In this study, systems analysis and population SNP analysis were performed to gain insight into the evolutionary trajectory of *G2-like* gene in maize expression patterns of some important *ZmG* genes were also investigated under cold and drought conditions.

## Materials and Methods

### Identification of *G2-like* genes

Maize protein and nucleic acid sequences were obtained from maize genome database (http://www.maizesequence.org). *S*orghum data were downloaded from Phytozome (v9.1). DNATOOLS software was used to construct a local database of maize nucleotide and protein sequences. Previously reported sequences of *Arabidopsis* G2-like protein sequences were used as queries to search against the maize and sorghum protein database with BLASTP (E-values below 0.001). All candidate sequences that met the standards were confirmed to be G2-like proteins using Pfam (http://pfam.xfam.org/) and SMART (http://smart.embl-heidelberg.de/). Finally, sequences of all confirmed proteins were aligned using MEGA6 software [29], and redundant sequences were removed manually (all sequence data in supplementary file). Physical parameters of these proteins including molecular mass (kDa), and isoelectric point (pI) were estimated using the compute pI/Mw tool in ExPASy (http://web.expasy.org/compute_pi/). Protein subcellular localization was predicted by online softwares: TargetP online server (http://www.expasy.org/), SubLoc online server (http://www.bioinfo.tsinghua.edu.cn/SubLoc/) and WoLF PSORT online server (http://www.genscript.com/).

### Phylogenetic and motif analysis of *G2-like* genes

A neighbor-joining phylogenetic tree of *G2-like* genes from maize and sorghum was generated with MEGA6 software [29]. The confidence limits of each branch were assessed by 1,000 bootstrap replications and expressed as percentage values.

Conserved motifs among maize *G2-like* genes were examined using MEME software (http://meme-suite.org/) [30]. The width of each motif was limited to 6–50, and the maximum number of motifs was set to 20. In addition, structural motif annotation was performed using Pfam (http://pfam.xfam.org/) and SMART (http://smart.embl-heidelberg.de/) online service.

### Chromosomal locations, gene structure and duplication events of *G2-like* genes

*G2-like* genes were mapped to the maize chromosomes according to their position information from maize genomic database (http://www.maizesequence.org). MapInspect software (http://www.plantbreeding.wur.nl/uk/software_mapinspect.html) was subsequently used to graphically portray *G2-like* genes from maize. Introns and exons were predicted via the online tool Gene Structure Display Server 2.0 (http://gsds.cbi.pku.edu.cn/) [31].The duplication pattern for each *G2-like* genes was analyzed by MCScanX softaware (http://chibba.pgml.uga.edu/mcscan2/) [32]. Then, whole-genome BLASTP analysis of maize and sorghum were performed using local blast software with e-value under 1e-5. Blast outputs and position of all protein-coding genes were imported into Circos software (http://circos.ca/) [33] and genes were classified into various types of duplications.

The Ka and Ks were calculated by DnaSPv5.0 [34]. The ratio of nonsynonymous to synonymous nucleotide substitutions (Ka/Ks ratios between paralogs) was analyzed to detect the mode of selection. Values of Ka/Ks >1, = 1 and <1 represent positive selection, neutral selection and purifying selection, respectively [35].

### Expression pattern analysis

Gene expression data from Maize B73 transcriptomes were used to draw a heat map, including germinating seeds, primary roots, stems, SAMs (shoot apical meristem), leaves, endosperm, embryos and whole seeds [36,37]. The expression data were used to generate a heatmap using R/Bioconductor (http://www.bioconductor.org/).

### RNA extraction and qRT-PCR analysis

The maize inbred line B73 was grown in a greenhouse (16h light/ 8h dark, 28±2°C). After 3 weeks, seedlings were treated with cold and drought stresses, respectively. For cold stress, leaves were sampled at 0h, 6h, 12h and 24h after cold stress (4°C) treatment. For drought stress, leaves were collected at 0h, 0.5h, 1h, 2h, 3h and 6h after treated with 20% PEG6000. Total RNAs of all the samples collected in this study were extracted using the RNAiso plus (TaKaRa) accords to the manufacturer’s instructions. The DNase-treated RNA was reverse-transcribed using First-Strand cDNA Synthesis Kit (TRANSGEN). Reaction was used SYBR Green Master Mix reagent (Roche). The qRT-PCR was performed in a 20 μl volume, which contained 10 μl of 2×SYBR Green Master Mix, 2 μl diluted cDNA template, 1 μl of each specific primer, and 6 μl ddH2O and then performed on ABI 7300 real time system (Applied Biosystems). The qRT-PCR program was used as follows: 95°C for 10 min, followed by 40 cycles at 95°C for 15 s and 60°C for 1 min. The gene-specific primers designed by Primer 6.0 software were employed to amplify 120–300 bp PCR products unique to each gene (S7 Table). Expression level of the maize *Actin 1* gene was used as an internal control. The relative expression level of each gene was calculated as 2^*-ΔΔCT*^ [38] compared to that of untreated control plant which was set as 1. The qRT-PCR assays were performed with three biological and three technical replicates.

### Nucleotide diversity of *G2-like* genes in 85 maize inbreed lines

To further investigate *G2-like* gene evolution in natural populations of maize, *G2-like* genes sequences were examined from 85 maize inbred lines grown in tropical and temperate regions combined with whole genome sequencing SNP data (unpublished data). DNASP 5.0 software was used to analyze sequence nucleotide polymorphism and gene flow. The nucleotide diversity parameter Pi (π) was estimated, where Pi is the average number of nucleotide differences per site between any two DNA sequences. In addition, in gene flow analysis, G_st_ is the genetic differentiation coefficient and F_st_ is the fixed coefficient. χ^2^ assessment was used to test the significance of genetic differences among groups.

## Results

### Identification and analysis of *G2-like* genes in maize

After extensive searches and alignment of the maize genome database using previously reported *Arabidopsis* G2-like proteins AtGLK1 (AT2G20570) and AtGLK2 (AT5G44190) [23] as BLASTP queries, a total of 59 G2-like genes (designated *ZmG1* to *ZmG59*) were identified (S1 Table and S1 Text). Basic information about maize G2-like genes is presented in S1 Table. The exon number of these 59 genes ranges from 1 to 8. The predicted molecular weights (MW) of ZmG proteins range from 16.68 kDa to 59.22 kDa, while their lengths range from 145 to 554 amino acids. ZmG10 has the shortest sequence while ZmG37 has the longest. In addition, the proteins could be divided into two groups of roughly equal size based on isoelectric point, comprising 30 acidic proteins and 29 basic proteins. Moreover, the subcellular localizations of 59 genes predicted by the three online server TargetP online server (http://www.expasy.org/), SubLoc online server (http://www.bioinfo.tsinghua.edu.cn/SubLoc/) and WoLF PSORT online server (http://www.genscript.com/). Combined with results from the three online server analyses, we found that most of ZmGs were predicted in the nucleus.

### Phylogenetic and motif analysis of maize *G2-like* genes

**Fig 1:**
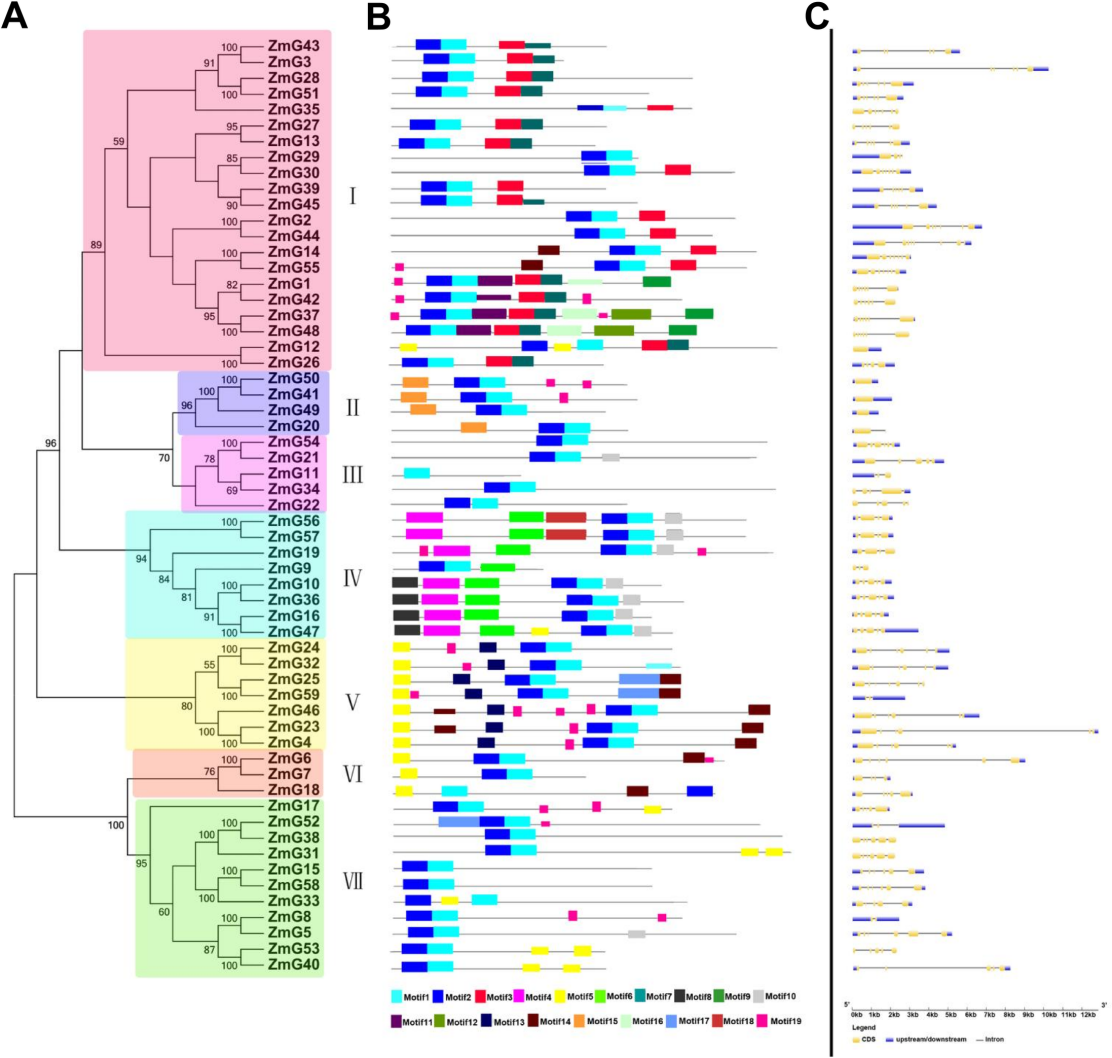
Neighbor-joining phylogenetic tree, motif and structure of the 59 predicted maize *G2-like* genes. (A) The phylogenetic tree, constructed with MEGA6.0, was generated based on amino acid sequences. G2-like proteins were divided into seven groups (I-VII) based on high bootstrap values (>50). (B) All motifs were identified by MEME using the complete amino acid sequences of 59 maize G2-like proteins. Different motifs are indicated by different-colored numbers (1–20) as shown in the scale at the bottom of the Fig (C) Exons and introns are indicated by yellow boxes and single lines, respectively. Thick blue lines represent untranslated regions (UTRs). The length of each *G2-like* gene can be estimated based on the scale at the bottom.

The phylogenetic relationships and evolutionary history of maize *G2-like* gene family were inferred by constructing a phylogenetic tree based on aligned G2-like protein sequences (Fig 1A). The *G2-like* family was classed into seven groups (I–VII) based on evolutionary relationships and motif analysis; Groups I to VII contain 21, 4, 5, 8, 7, 3 and 11 genes, respectively.

Motif analysis of 59 maize *G2-like* genes also proved phylogenetic kinship. Conserved motifs (Fig 1B and S2 Table) were examined using MEME software and the motif sequences were shown in S2 Table. In addition to a conserved G2-like Myb DNA-binding domain, each group has unique motifs that might represent their functional diversity. Group I possess a Myb-CC-LHEQLE domain (a type of MYB-like domain). MYB transcription factors play diverse roles in plant development and in response to abiotic stress [39]. Besides, in gene structure analysis diverse distribution of intronic regions (from 1 to 8 in numbers) was found among *ZmG* genes (Fig 1C). In general, *ZmGs* clustered in the same group exhibit similar exon/intron structure (Fig 1C) and all *ZmGs* genes in group Ⅱ have no intron.

Multiple sequence alignment of ZmGs and other identified G2-like proteins of rice OsGLK1/2 (LOC_Os06g24070/ LOC_Os01g13740) and *Arabidopsis* AtGLK1/2 (AT2G20570/ AT5G44190) [23] (S1 Fig) demonstrated that these sequences are particularly conserved across two regions of a putative DNA binding domain with an HLH structure (The first helix contains initial sequences PELHRR and invariably comprises 14 amino acids and the second helix contains an initial NI/VASHLQ motif. These helices are separated by a 22 amino acid loop). In a number of well-characterized transcriptional regulators, HLH domains bind DNA and mediate dimerization [40,41].

Moreover, to investigate the potential function of maize *G2-like* genes, further evolutionary relationship among *ZmGs*, *AtGLK1*, *AtGLK2*, *OsGLK1* and *OsGLK2* was investigated. As showed in S2 Fig, *AtGLK1*, *AtGLK2*, *OsGLK1* and *OsGLK2* were classed into one group (*ZmG54* classed with *OsGLK1*, and *ZmG21* classed with *OsGLK2*).

### Physical locations and duplication events of *G2-like* genes in maize

**Fig 2:**
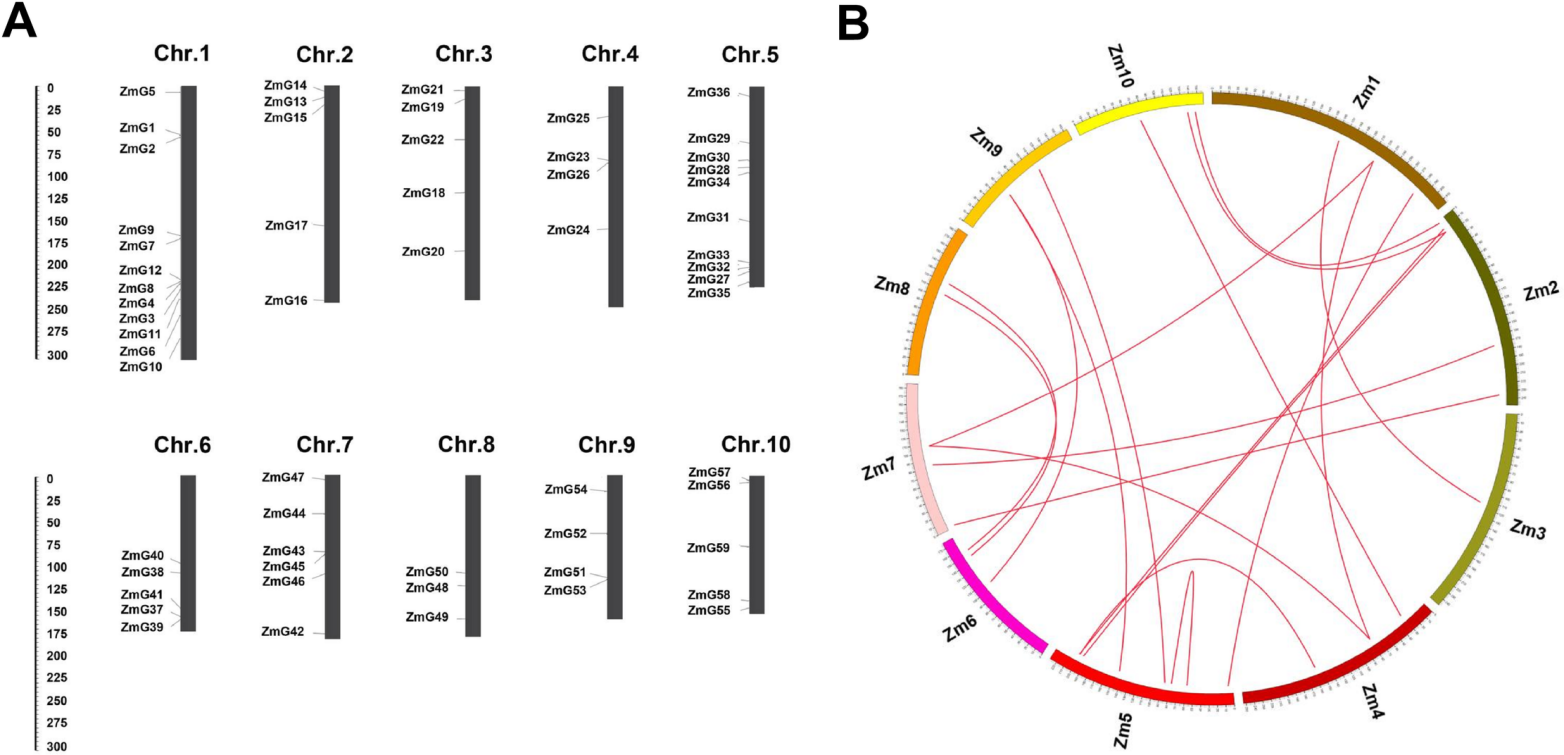
Locations and duplication events of maize *G2-like* genes. (A) Chrosome locations of maize G2-like genes. The scale represents megabases (Mb). The chromosome numbers are indicated above of each bar. **(**B) Synteny of maize *G2-like* gene. Numbers along each chromosome box indicate sequence lengths in megabases. All the syntenic genes were located in map, and linked by red lines.

Chromosome locations of maize 59 *G2-like* genes revealed that 11, 5, 5, 4, 10, 5, 6, 3, 5 and 5 genes are distributed on chromosomes 1 to 10, respectively (Fig 2A). Although *G2-like* genes are distributed on every maize chromosome, their distribution and gene groups are nonrandom. For example, chromosome 1 not only contains the greatest number of *G2-like* genes (11), but it also exhibits the greatest variation of gene groups, whereas only three *G2-like* genes are located on chromosome 8. In addition, 18 duplicated pairs of *ZmG* genes were identified in syntney map (Fig 2B), and all of them were segmental duplication events. *ZmG*s duplication events might have resulted from ancient tetraploidy processes during the course of evolution. All chromosomes were involved in these duplication events.

The Ka/Ks ratio (ratio of nonsynonymous to synonymous nucleotide substitutions) is used as an indicator of selective pressure acting on a protein-coding gene [42]. The Ka (number of nonsynonymous substitutions per nonsynonymous site) and Ks (number of synonymous substitution per synonymous site of duplicated genes) values of 18 *ZmG*s pairs were calculated (Table 1), and results showed that five pairs genes were undergone positive selection (Ka/Ks> 1), and others were purifying selection (Ka/Ks< 1).

**Table 1:** Ka/Ks and divergence analysis of paralogous maize G2-like genes.

Paralogoue pair	Ka	Ks	ka/ks	Selection pressure	Duplicate type
*ZmG55/13*	1.01	1.01	1.01	Positive selection	Segmental duplication
*ZmG50/27*	0.99	1.07	1.08	Positive selection	Segmental duplication
*ZmG45/4*	0.96	1.15	1.19	Positive selection	Segmental duplication
*ZmG35/9*	0.75	0.94	1.26	Positive selection	Segmental duplication
*ZmG4/22*	0.69	0.99	1.43	Positive selection	Segmental duplication
*ZmG12/26*	1.06	0.90	0.85	Purifying selection	Segmental duplication
*ZmG36/47*	0.56	0.46	0.82	Purifying selection	Segmental duplication
*ZmG28/29*	1.18	0.92	0.78	Purifying selection	Segmental duplication
*ZmG27/7*	0.92	0.85	0.93	Purifying selection	Segmental duplication
*ZmG23/31*	1.00	0.91	0.91	Purifying selection	Segmental duplication
*ZmG58/14*	1.23	0.99	0.80	Purifying selection	Segmental duplication
*ZmG32/14*	0.96	0.77	0.80	Purifying selection	Segmental duplication
*ZmG22/45*	1.39	0.97	0.70	Purifying selection	Segmental duplication
*ZmG15/46*	0.99	0.88	0.89	Purifying selection	Segmental duplication
*ZmG24/59*	0.69	0.58	0.83	Purifying selection	Segmental duplication
*ZmG37/51*	2.09	0.58	0.28	Purifying selection	Segmental duplication
*ZmG49/40*	1.02	0.95	0.93	Purifying selection	Segmental duplication

### Phylogenetic comparison of *G2-like* genes between maize and sorghum

**Fig 3:**
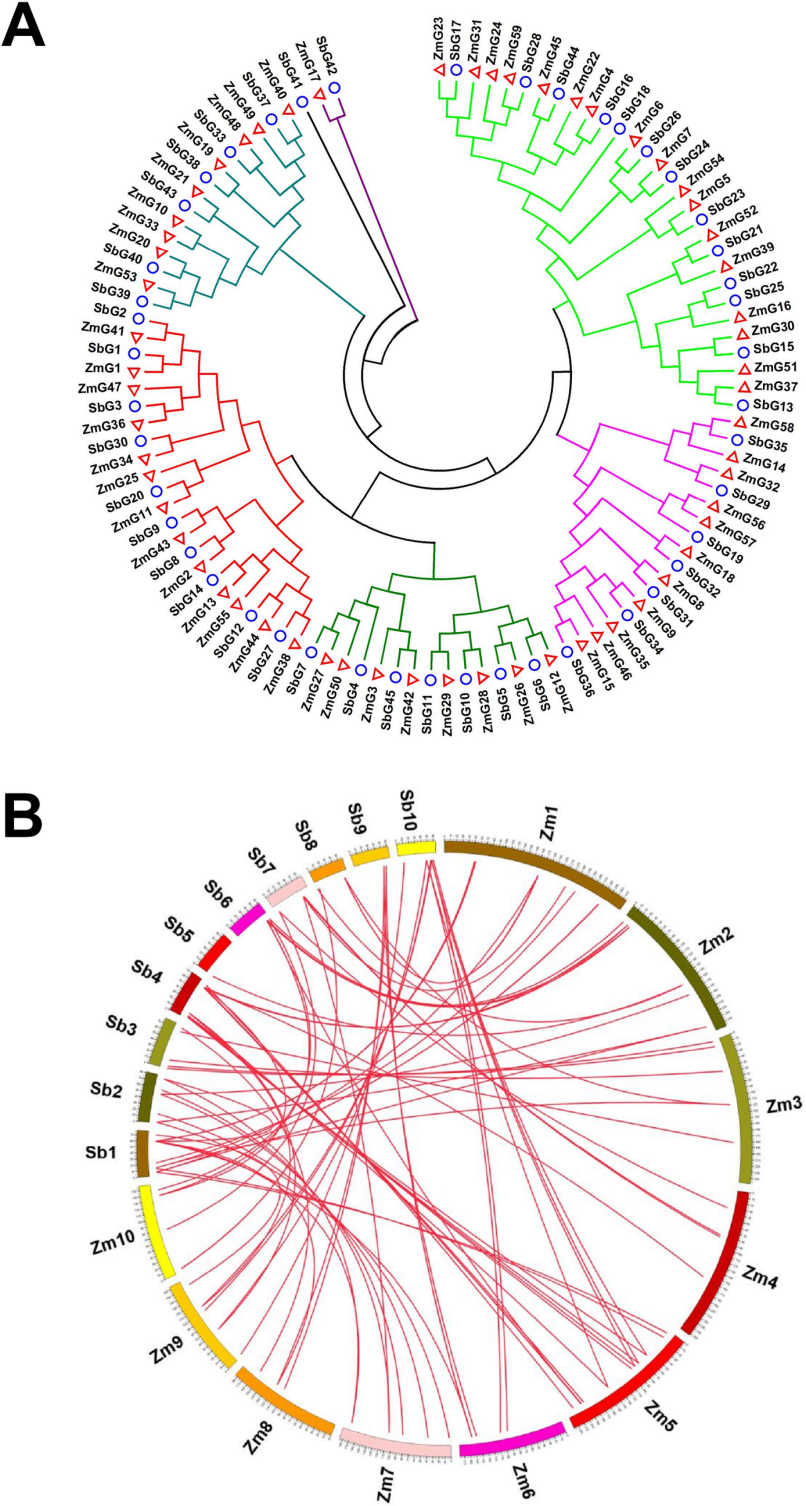
Phylogenetic analysis and duplication events of G2-like proteins between maize and sorghum. (A) Phylogenetic tree of *ZmG*s and *SbG*s. The tree was constructed using neighbor-joining method with MEGA 6.0. Maize G2-like proteins were presented by hollow red triangle, and sorghum G2-like proteins were presented hollow blue circular. **(**B) Synteny of maize and sorghum G2-like genes regions. Numbers along each chromosome box indicate sequence lengths in megabases. Maize and sorghum chromosome were presented by Zm1-Zm10 and Sb1-Sb10, respectively. All the syntenic genes across maize and sorghum were linked by red lines.

To further analyze evolutionary relationship of *G2-like* family, *ZmG*s and *SbG*s (G2-like genes of sorghum) were subjected to a comprehensive phylogenetic analysis. 45 sorghum *G2-like* genes (*SbG1*-*SbG45*) were identified (S3 Table and S2 Text), and 11 collinear gene pairs were found (S4 Table), distributing in 9 chromosomes (except chromosome 3) (S3 Fig). Then the unrooted phylogenetic tree between *ZmG*s and *SbG*s was constructed using the full-length protein sequences (Fig 3A). The phylogenetic analysis classified the *ZmG*s into several groups together with their sorghum orthologs. To identify the evolutionary orthologous relationships within *G2-like* genes of maize and sorghum, a synteny map was plotted between maize and sorghum (Fig 3B). A total of 75 orthologous gene pairs between maize and sorghum were found (S5 Table). Across maize and sorghum, strongly conserved microsyntny among regions hosting *G2-like* genes were observed, especially in Zm1 and Sb1 (6 synteny genes), Zm3 and Sb3 (4 synteny genes), Zm5 and Sb10 (4 synteny genes), Zm5 and Sb4 (9 synteny genes), Zm7 and Sb2 (6 synteny genes), Zm9 and Sb10 (4 synteny genes).

### Digital expression analysis

**Fig 4:**
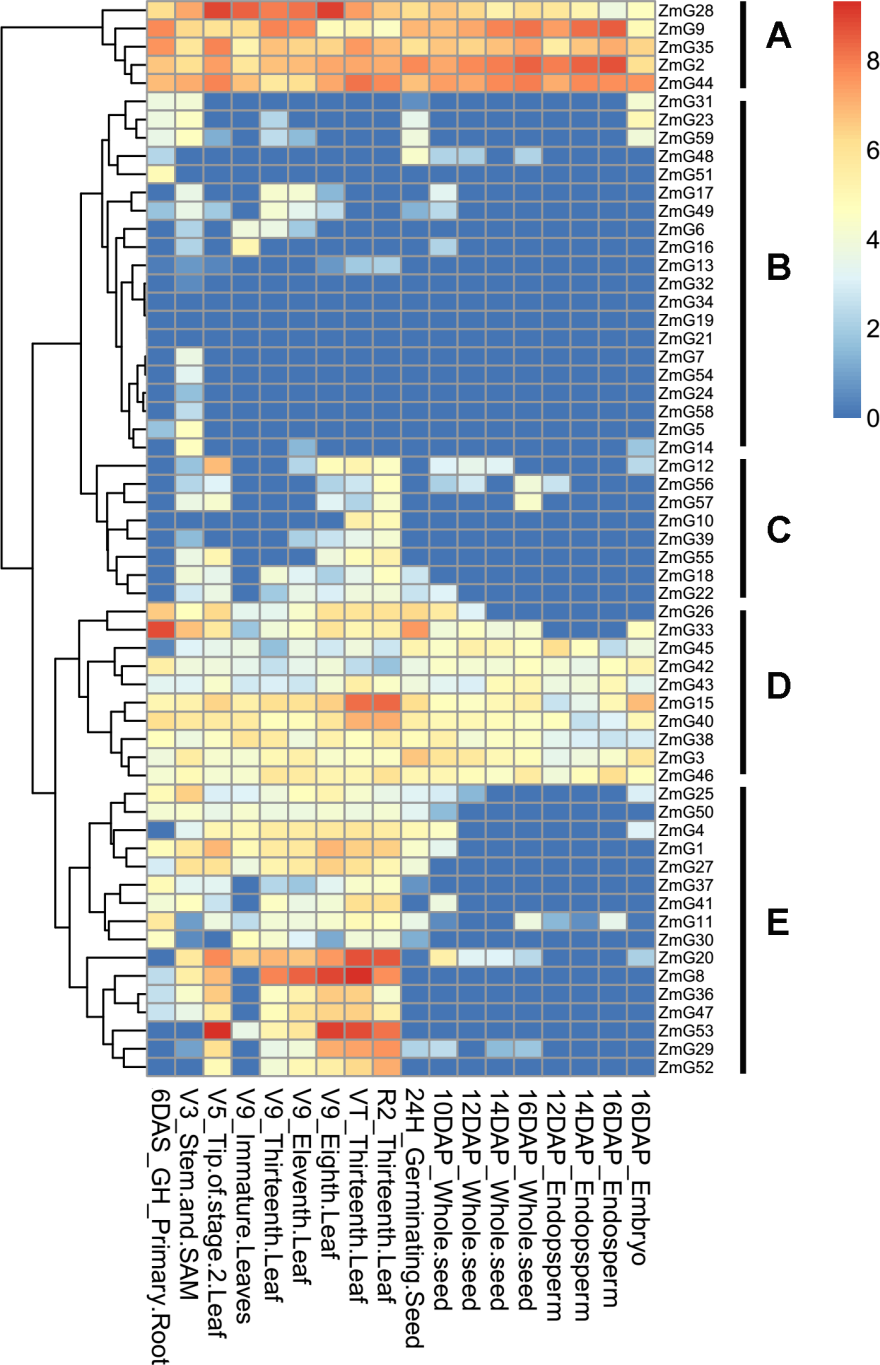
Heat map of maize G2-like genes. The expression level of each *G2-like* gene can be estimated based on the scale to the right. Red, yellow and blue indicate high, medium and low levels of gene expression, respectively. H, hours; DAS, days after sowing; GH, greenhouse; V, vegetative; DAP, days after pollination; VT, vegetative tasseling; R, reproductive.

Gene expression patterns in different tissues provide important information for studying gene function. Results of expression pattern analysis (Fig 4) revealed that most *G2-like* genes were expressed in green tissues (e.g., stems and leaves), but some genes were expressed in non-green tissue (e.g., seeds, roots endosperm and embryos). The gene family consisted of five groups based on expression patterns (A–E). Pattern A and pattern D genes were expressed during almost all periods in seed, root, stem, leaf, endosperm and embryo tissue, with high levels of expression, especially for pattern A genes. Pattern B genes exhibited little or no expression in any tissue or organ, with only a few genes expressed in stems and leaves during some periods. Pattern C and E genes were mainly expressed in leaves, with high expression levels. We also examined the expression patterns of duplicated groups of G2-like genes. Pattern B contains the most duplicated genes (11 of 30 duplication genes). Pattern A, C and D include only 1, 2 and 3 duplicated genes, respectively, while pattern E contains 8 duplicated genes. Among these duplicated gene pairs, eight pairs (16 genes) shared the same expression patterns, including the following: *ZmG9* and *ZmG35* (pattern A); *ZmG23* and *ZmG31*, *ZmG24* and *ZmG59*, *ZmG58* and *ZmG14*, *ZmG32* and *ZmG58* (pattern B); *ZmG15* and *ZmG46* (pattern D); and *ZmG36* and *ZmG47*, *ZmG50* and *ZmG27* (pattern E).

### Expression levels of maize *G2-like* genes in response to cold and drought stress

**Fig 5:**
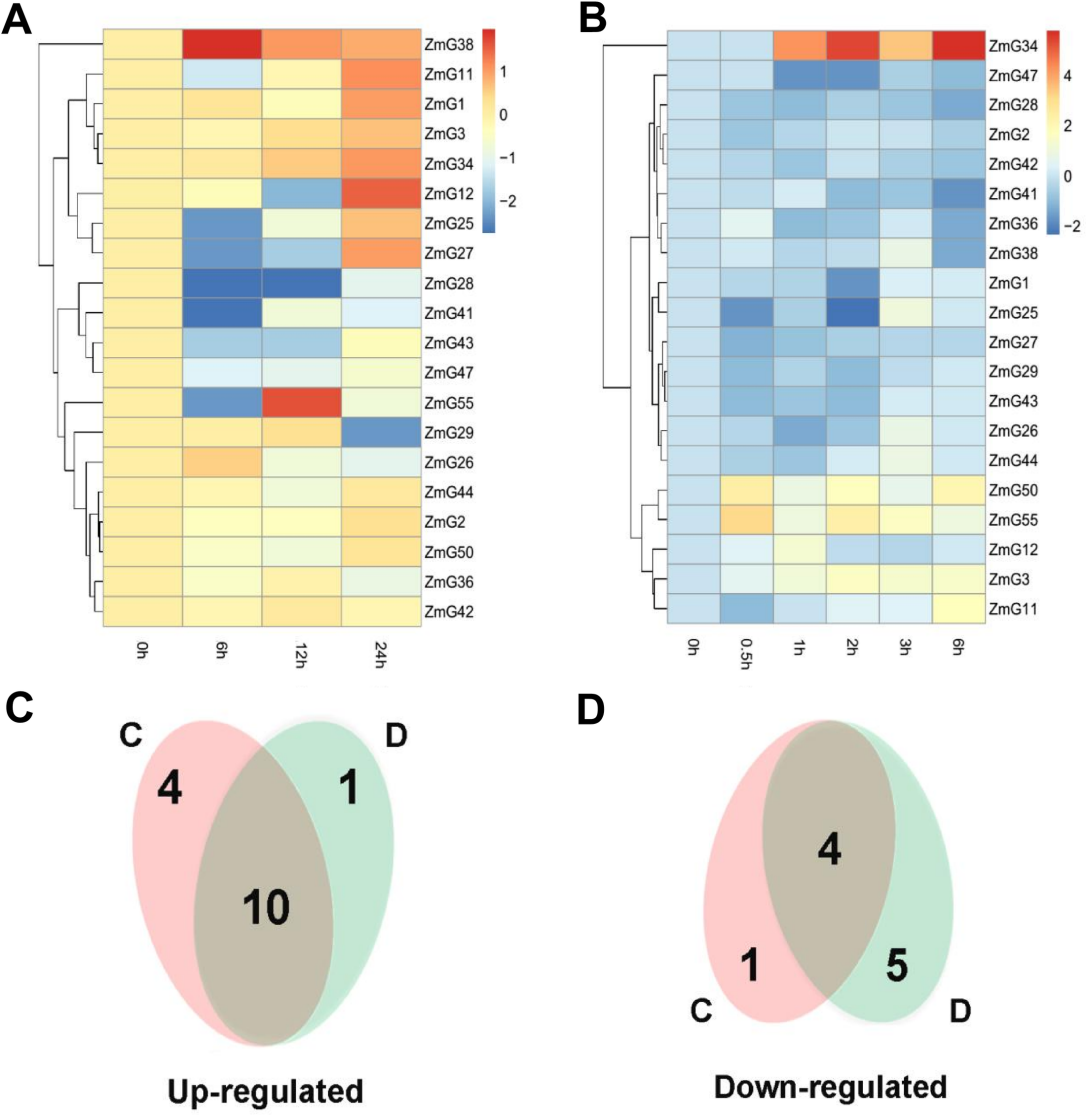
Expression of twenty maize stress-responsive *G2-like* genes under stress treatments. Three-week-old seedlings were treated with 20% PEG6000 (A) and 4°C (B), respectively. Relative expression levels of the *ZmG* genes were analyzed by quantitative qPCR, and log_2_-transformed fold-change values were used for creating the heatmap (original data were shown in S4 Fig). Venn diagram illustrated the distribution of the up-regulated (C) or down-regulated (D) *ZmG* genes response to cold and drought treatments. The common subset of genes regulated by the two stressed was marked by the overlapping circle.

In this study, we investigated possible stress-responsive *cis*-elements in the promoter regions of these genes. Two abiotic relative *cis*-elements DRE (dehydration-responsive element) and LTRE (low-temperature responsive element) were detected in these genes 2000 bp promoter sequences, which may be responsive to their stress responsiveness (Table 2). Then we further investigated expression levels of some *G2-like* genes in response to abiotic stress by subjecting three-week-old seedling leaves to drought (20% PEG6000) and cold (4°C) treatments. We choose some *ZmG* genes for the followed verification experiment-qRT-PCR, randomly. Detailed expression profiles of these *G2-like* genes under cold and drought stress conditions were presented in S4 Fig. Heat map representation for transcript expression fold change in response to these two abiotic stresses was shown in Fig 5. Ten genes (*ZmG3*, *ZmG11*, *ZmG12*, *ZmG25*, *ZmG26*, *ZmG34*, *ZmG38*, *ZmG44*, *ZmG50* and *ZmG55*) were up-regulated by both drought (Fig 5A) and cold stresses (Fig 5B), while four genes (*ZmG28*, *ZmG41*, *ZmG43* and *ZmG47*) were down-regulated under both two conditions. Moreover, the expression of five genes (*ZmG1*, *ZmG2*, *ZmG27*, *ZmG29* and *ZmG42*) were induced (Except *ZmG42*) by drought stress (Fig 5A) but repressed by cold (Fig 5B), and only one gene (*ZmG36*) was induced by cold but repressed by drought.

**Table 2:** *Cis*-elements in the promoter regions of stress-responsive G2-like genes.

Genes No.	Promotor Elements		Genes No.	Promotor Elements	
	DRE	LTRE		DRE	LTRE
*ZmG1*	2	3	*ZmG31*	0	2
*ZmG2*	2	2	*ZmG32*	1	1
*ZmG3*	0	6	*ZmG33*	0	0
*ZmG4*	7	9	*ZmG34*	1	2
*ZmG5*	0	1	*ZmG35*	0	1
*ZmG6*	0	0	*ZmG36*	3	3
*ZmG7*	8	7	*ZmG37*	0	1
*ZmG8*	0	2	*ZmG38*	0	1
*ZmG9*	0	9	*ZmG39*	3	4
*ZmG10*	0	1	*ZmG40*	2	2
*ZmG11*	2	2	*ZmG41*	0	0
*ZmG12*	9	0	*ZmG42*	4	4
*ZmG13*	2	4	*ZmG43*	1	2
*ZmG14*	1	3	*ZmG44*	2	6
*ZmG15*	1	1	*ZmG45*	0	2
*ZmG16*	2	4	*ZmG46*	0	0
*ZmG17*	1	1	*ZmG47*	1	4
*ZmG18*	2	2	*ZmG48*	0	0
*ZmG19*	0	0	*ZmG49*	4	3
*ZmG20*	6	5	*ZmG50*	0	2
*ZmG21*	0	0	*ZmG51*	0	0
*ZmG22*	3	4	*ZmG52*	1	1
*ZmG23*	1	1	*ZmG53*	0	3
*ZmG24*	1	2	*ZmG54*	7	6
*ZmG25*	1	2	*ZmG55*	1	0
*ZmG26*	8	6	*ZmG56*	0	0
*ZmG27*	1	3	*ZmG57*	0	4
*ZmG28*	0	1	*ZmG58*	0	5
*ZmG29*	0	1	*ZmG59*	0	0
*ZmG30*	0	1			

### Nucleotide diversity of *G2-like* genes in 85 maize inbreed lines

**Fig 6:**
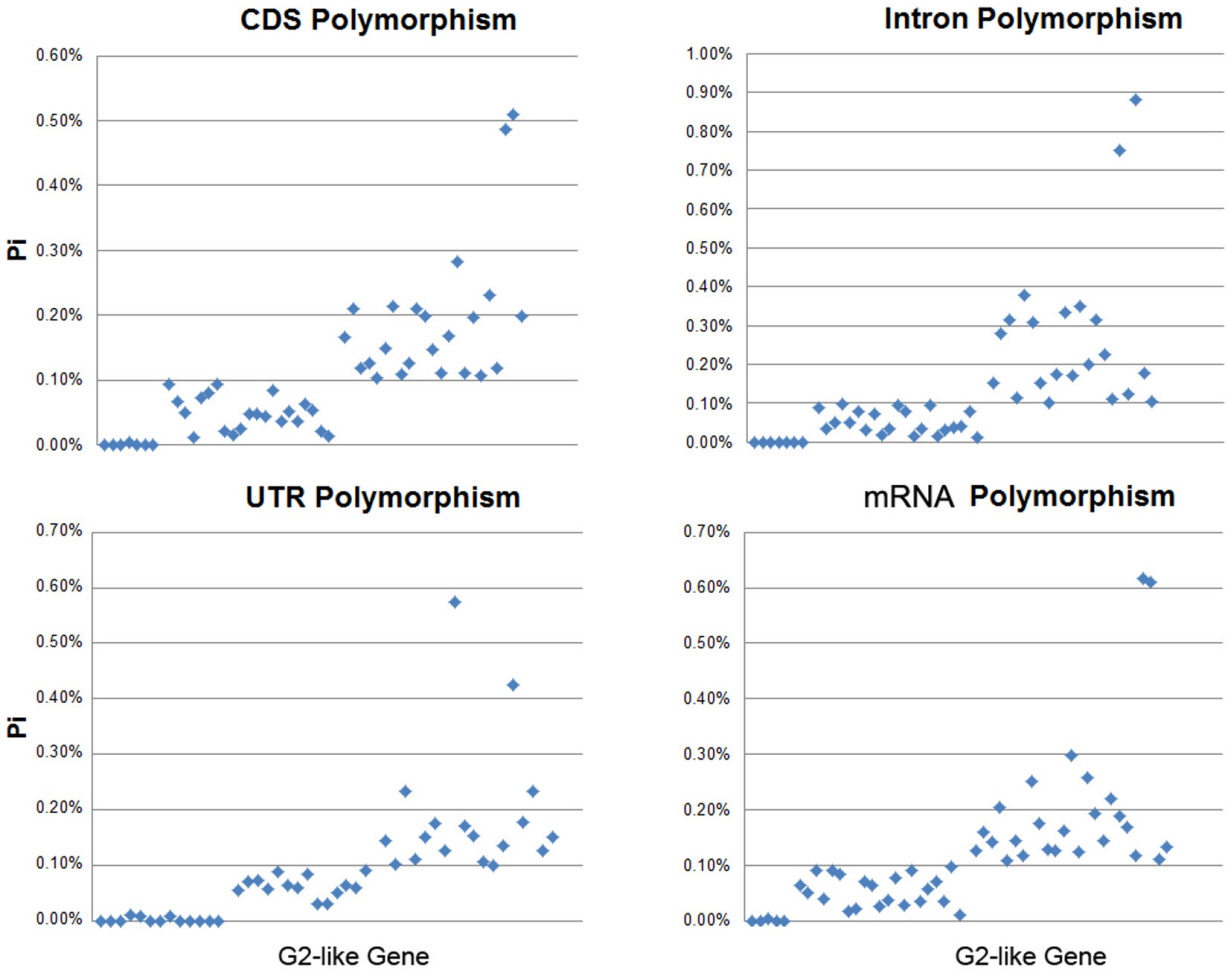
Polymorphism of *G2-like* genes. The polymorphism in different conserved regions (CDS, UTR, intron and mRNA) is shown. Vertical axis shows the nucleotide polymorphism Pi, and the horizontal axis represents *G2-like* genes in maize.

The polymorphism of *G2-like* genes in different regions (CDS, UTR, introns and mRNA) among 85 maize inbreed lines were further analyzed (Fig 6). Categories of *ZmG*s polymorphism on all regions were divided depending on Pi value (Pi is the average number of nucleotide differences per site between any two DNA sequences). Results showed that these four regions could be classified into similar groups: Pi ≤ 0.01%, 0.01% < Pi ≤ 0.1% and Pi > 0.1%, which we designated Type I, Type II and Type III, respectively. Type III includes the largest genes, while Type I, harboring a low level of nucleotide variation, and includes the fewest genes. We analyzed the Ka/Ks ratios of highly diverse genes among 85 maize lines (Table 3) to explore the selection pressure they are undergoing. Among the 23 genes examined, only one gene (*ZmG34*) was under positive selection.

**Table 3:** Ka/Ks and selection pressure on diverse G2-like genes.

Gene ID	Ka	Ks	Ka/Ks	selection Pressure
ZmG1	0.000796	0.002883	0.275923	Negative selection
ZmG5	0.001525	0.003635	0.419355	Negative selection
ZmG6	0.000968	0.003109	0.311423	Negative selection
ZmG9	0.001677	0.006709	0.249946	Negative selection
ZmG11	0.000879	0.001751	0.50206	Negative selection
ZmG14	0.000763	0.002921	0.261349	Negative selection
ZmG15	0.001667	0.00301	0.553781	Negative selection
ZmG17	0.001898	0.003724	0.509689	Negative selection
ZmG24	0.000871	0.001564	0.556699	Negative selection
ZmG26	0.001311	0.004799	0.273232	Negative selection
ZmG27	0.000316	0.006472	0.048882	Negative selection
ZmG30	0.002397	0.01413	0.169648	Negative selection
ZmG33	0.000548	0.002577	0.212772	Negative selection
ZmG34	0.005853	0.002575	2.273277	positive selection
ZmG35	0.001462	0		
ZmG37	0.00091	0.002306	0.394459	Negative selection
ZmG38	0.00016	0.007295	0.021897	Negative selection
ZmG39	0.001992	0		
ZmG41	0.000674	0.005051	0.133499	Negative selection
ZmG46	0.00076	0.006038	0.125846	Negative selection
ZmG53	0.000275	0.003129	0.08802	Negative selection
ZmG54	0.000958	0.002034	0.470698	Negative selection
ZmG58	0.001525	0.003635	0.419355	Negative selection

### Comparison of *G2-like* genes genetic diversity between tropical and temperate lines

Nucleotide polymorphism of G2-like genes among 85 tropical and temperate maize inbred lines were also examined (S6 Table). The single nucleotide polymorphisms (SNP) between tropical and temperate lines were not significantly different, whereas, genetic variance analysis between these lines revealed that 14 genes (27.45%) exhibited differences between tropical and temperate lines, among which 11 genes (*ZmG37*, *ZmG47*, *ZmG14*, *ZmG39*, *ZmG32*, *ZmG11*, *ZmG15*, *ZmG22*, *ZmG17*, *ZmG30* and *ZmG5*) exhibited significant differences (P < 0.01). We further analyzed the haplotype diversity between tropical and temperate lines (S6 Table), revealing that the haplotype diversity of tropical lines is greater than temperate. Seven of the 11 genes had moderate genetic differentiation, with Fst values (fixation index of the subpopulation within the total) [43] between 0.05 and 0.15, while 4 other genes (*ZmG22*, *ZmG11*, *ZmG32* and *ZmG47*) had a high level of genetic differentiation, with Fst values greater than 0.15.

**Fig 7:**
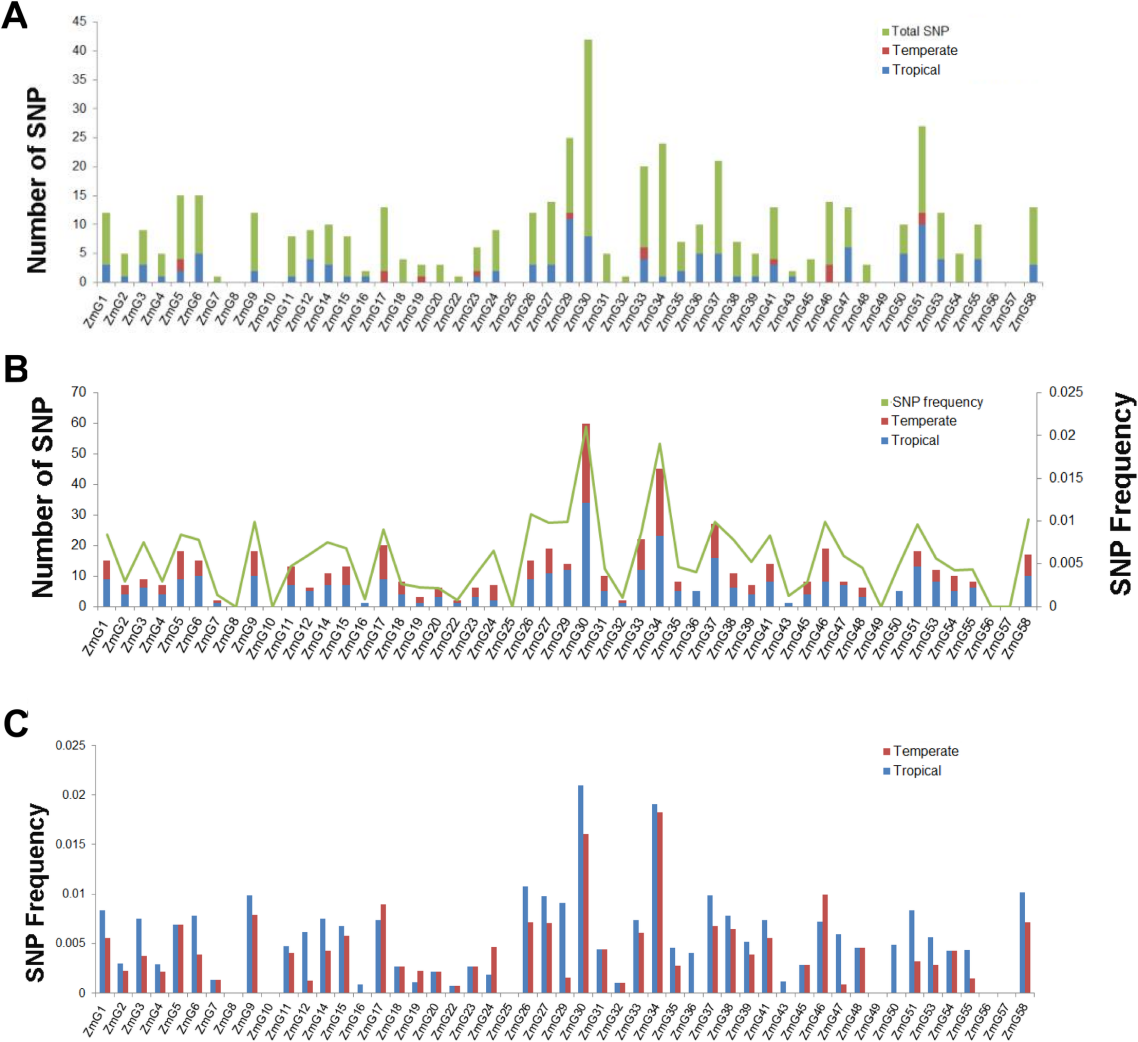
Number of SNPs in each *G2-like* gene (CDS sequence) among 85 maize inbred lines. Blue boxes represent the number of SNPs in tropical lines, red boxes represent the number of SNPs in temperate lines, green boxes represent the total number of SNPs and green lines indicate SNP frequency. (A) Number of tropical and temperate fixed SNPs and total number of SNPs in 85 maize inbred lines. (B) Number of SNPs in G2-like genes in tropical and temperate lines and SNP frequency. (C) Comparisons of tropical and temperate lines SNP frequency.

We compared the number of unique fixed SNPs in each G2-like gene in the tropical and temperate lines, respectively (Fig 7A). Results showed that *G2-like* genes of tropical lines have much more unique fixed SNP sites compared to temperate lines. Only five genes (*ZmG5*, *ZmG23*, *ZmG17*, *ZmG19* and *ZmG46*) have as many or more unique fixed SNP sites in temperate versus tropical lines. However, the total number of fixed SNP statistics showed that *ZmG* genes shared more same fixed SNPs than their unique fixed SNPs in both tropical and temperate lines (Fig 7A), suggesting that these SNPs may be not selected based on the environment relative to temperature. Furthermore, the total number of fixed SNPs in tropical and temperate lines was analyzed (Fig 7B), revealing that more fixed SNPs of tropical lines are present than temperate lines. The frequency of fixed SNP exhibited variation that is always proportional to the number of SNPs per gene (the SNP frequency is the ratio of the total SNP number of each gene with their corresponding coding sequence length) (Fig 7B). However, 42 of 59 *ZmG* genes in tropical lines have higher fixed SNP frequency than temperate lines (Fig 7C).

## Discussion

Interestingly, in this study, 59 *G2-like* genes were identified in maize and a same set of *G2-like* gene was identified in GRASSIUS (http://grassius.org/grasstfdb.html) database ever (S3 Text). And *ZmG* genes in this study matched the list in GRASSIUS, except for one (*ZmG34* and *ZmGLK12*). We checked these two genes, and found that *ZmGLK12* does not contain Myb_DNA-binding domain, but *ZmG34* contains, indicating *ZmGLK12* was not *G2-like* gene.

In this study, numerous segmental duplications of maize *G2-like* genes were identified, indicating that segmental duplication was the main contributor to the expansion of maize *G2-like* genes. In tissues expression patterns, Pattern B contains the most duplicated genes (11 of 30 duplication genes), exhibited little or no expression in any tissue or organ, indicating some of these genes are pseudogenes or silenced paralogs. In general, segmental duplications are thought to occur regularly in more slowly evolving gene families. Thus we speculated that *G2-like* genes have been considerably conserved during the process of evolution. Since maize underwent whole genome duplication (WGD) event after diverging from sorghum, there ought to be twice as many *GLK* genes, but it is not double, clearly some have been lost 45 in sorghum versus 59 in maize. A phylogenetic comparison of *G2-like* genes between maize and sorghum divided 104 *G2-like* members into six clades, with *ZmG*s and *SbG*s appear to be more closely to each other. Moreover, synteny analysis showed, strongly conserved microsyntny among regions hosting *G2-like* genes across maize and sorghum was observed, further illustrated these genes derived from gene duplication.

The evolution of maize *G2-like* genes may have occurred during maize genome evolution. *G2-like* genes were amplified through duplication, which enabled C4 plants to undergo subfunctionalization, in turn enabling the development of cell-specific functions in dimorphic chloroplasts [44]. Current evidence suggests that the ancestral state was a single *G2-like* gene and that *G2-like* gene duplication enabled subfunctionalization [45]. Moreover, *G2-like* gene duplication preconditioned the evolution of chloroplast dimorphism. According to classical theory, when gene duplication occurs, each copy of the gene has two possible fates: (1) in most cases, a copy retains the original features, which are stabilized through negative selection; (2) the remaining copy does not undergo selection and becomes pseudogenic. This theory helps explain the slow evolution of *G2-like* genes and a certain level of total number of genes, further illustrating the highly conserved evolution of chloroplast. In rare cases, one copy of gene undergoes more adaptation, drive the evolution of gene function [46]. Based on analysis of *G2-like* gene family, we proposed that this family belongs to the former category, that is, the function of this gene family has been stabilized by duplication.

Furthermore, SNPs analysis of maize *ZmG* genes in natural population revealed the influences of temperature and humidity on *ZmG*s. Polymorphism analysis of *G2-like* genes in different conserved regions (Fig 6) revealed that highly diverse Type III includes the largest number of genes, which might have quickly evolved to adapt to environment, while highly conserved Type I includes the fewest genes, which may play an important role in basic functions rather than adaptation to temperature or humidity conditions. Selection pressure was revealed for genes in Type III, while only one gene (*ZmG34*) was under positive selection (Table 3), indicating that this family tends to retain vary in order to maintain stability. However, the variation retained in *ZmG34* might result from its significant role in growth and development in maize. And the expression pattern of *ZmG34* also suggested its importance. Genetic diversity analysis of *G2-like* genes between tropical and temperate lines (based on CDS) revealed 11 genes with Pi values lower than 0.01, which may be related to their role in the response to temperature and light conditions. These genes will be a focus for future research aimed at elucidating the influence of temperature and humidity conditions on maize growth. In these genes, *ZmG47* had a high level of genetic differentiation, and a high level expression in all tissues, we hypothesized that this gene may have significant roles in the formation and evolution of chloroplast.

In addition to total SNPs number, compared to temperate lines, more SNPs of *ZmG* genes were fixed in tropical lines. It suggested that these SNPs were selected based on the environment, which may relate to temperature and humidity. Our promoter element analysis results support this view, as promoters of these genes include DRE, ABRE, and LTRE resistance elements [47,48], and qRT-PCR results supported this view, too. In addition, a recent report suggested that *G2-like* genes play a role in the temperature stress response, as increased accumulation of *G2-like*1 was observed in frost-tolerant transgenic *Brassica napus* overexpressing two transcripts harboring DREB1/CBFs [49]. These stress-related genes are induced in order to adapt to environmental stresses. Overexpression of *AtGLK1* (*35S*: *AtGLK1*) in Arabidopsis confers resistance to the cereal pathogen *Fusarium graminearum* [27]. Thus, we speculate that maize *G2-like* gene family might have a function in stress and disease resistance. In addition, as *ZmG34* was significantly up-regulated by low temperature and drought stress, moreover, it was subjected to positive selection in natural population lines; we convinced more on this gene’s importance in crop breeding.

Due to highly conserved evolution of *G2-like* genes, we speculated these genes might play vital roles in maize growth and development. Moreover, both of these genes polymorphism and expression were sensitive for environment; further exploration of them would serve as useful information for maize culture in drought or cold environment.

## Supporting Information

S1 Fig *G2-like* genes sequences in maize, rice and Arabidopsis. Pileup multiple sequence alignment of maize ZmG1–59, rice OsGLK1–2 and Arabidopsis AtGLK1–2 GLK proteins. The putative DNA binding domain folds into an HLH structure. Black horizontal bars indicate the predicted α-helix segments conserved in all proteins. The GCT box is delimited, marked by red horizontal bars.
(TIF)

S2 Fig Phylogenetic relationship of maize G2-like proteins with the other G2-like proteins. (TIF)

S3 Fig Synteny of sorghum *G2-like* genes regions. Numbers along each chromosome box indicate sequence lengths in megabases. All the syntenic genes were located in sorghum chromosome, and linked by red lines.
(TIF)

S4 Fig Expression profile of *ZmG* genes in response to drought and cold stress. The Y-axis is the scale of relative expression levels. The X-axis is time courses of stress treatments. Error bars, +SE. (A) Relative expression levels of the twenty stress-responsive *G2-like* genes in responsive to drought stress. Seedlings were sampled at 0 h (CK), 6 h, 12 h, and 24 h after drought treatment. (B) Relative expression levels of the twenty stress- responsive G2-like genes in response to low temperature treatment (4°C). Seedlings were sampled at 0 h (CK), 0.5 h, 1 h, 2 h, 3 h and 6 h after 4°C treatment.
(TIF)

S1 Table Basic information about *G2-like* genes in maize. (DOC)

S2 Table Motif sequences identified using MEME tools. (DOCX)

S3 Table *G2-like* genes in Sorghum. (DOCX)

S4 Table Collinear gene pairs in sorghum. (DOCX)

S5 Table Orthologous gene pairs between maize and sorghum. (DOCX)

S6 Table Genetic diversity of *G2-like* genes between tropical (Tro) and temperate (Tem) inbred maize lines. (DOCX)

S7 Table qRT-PCR primers of maize *G2-like* genes. (DOCX)

S1 Text CDS, gene and protein sequences of maize *G2-like* genes in this study. (TXT)

S2 Text Protein sequences of sorghum *G2-like* genes in this study. (TXT)

S3 Text CDS and protein sequences of maize *GLK* genes in GRASS website. (TXT)
