# Supplementary figures and images for: Molecular Evolution and Genetic Variation of G2-Like Transcription Factor Genes in Maize
Source: PLoS One. 2016 Aug 25;11(8):e0161763. doi: 10.1371/journal.pone.0161763 (PMC4999087; doi:10.1371/journal.pone.0161763)

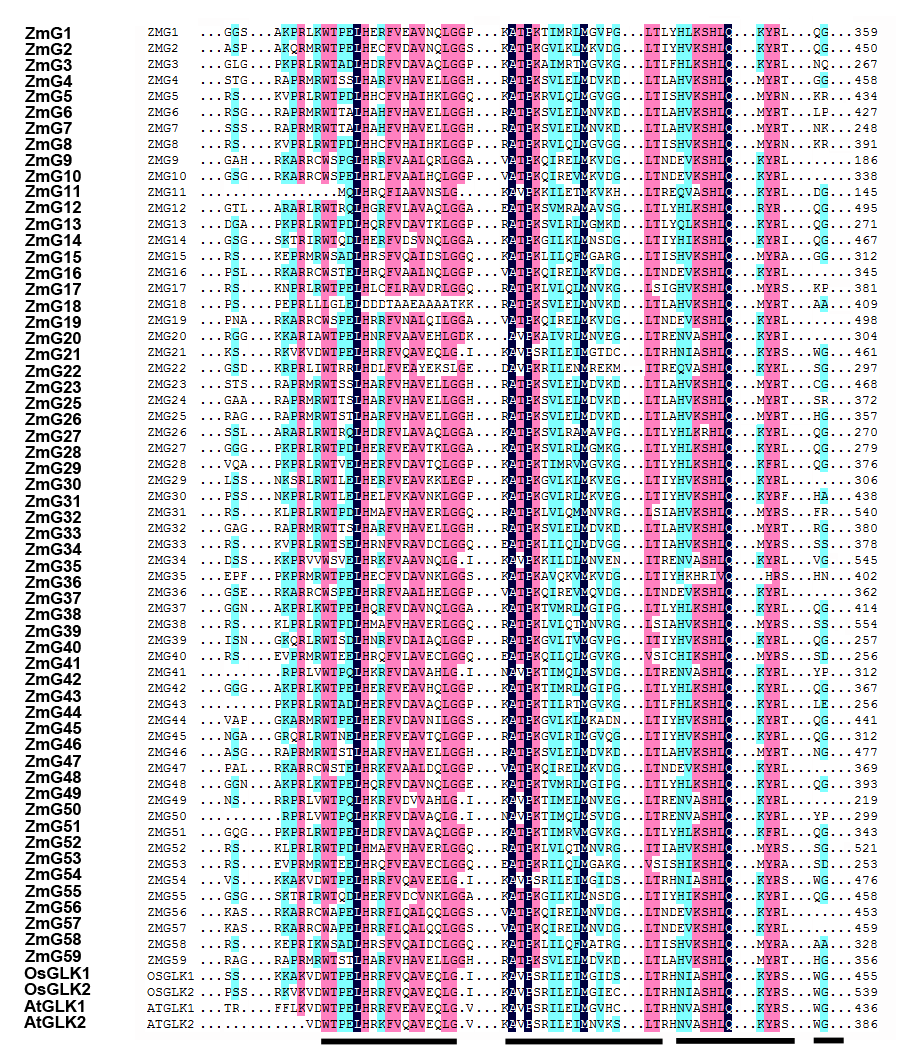

Supplement: S1 Fig — Pileup multiple sequence alignment of maize ZmG1–59, rice OsGLK1–2 and Arabidopsis AtGLK1–2 GLK proteins. The putative DNA binding domain folds into an HLH structure. Black horizontal bars indicate the predicted α-helix segments conserved in all proteins. The GCT box is delimited, marked by red horizontal bars. (TIF) [file pone.0161763.s001.tif]

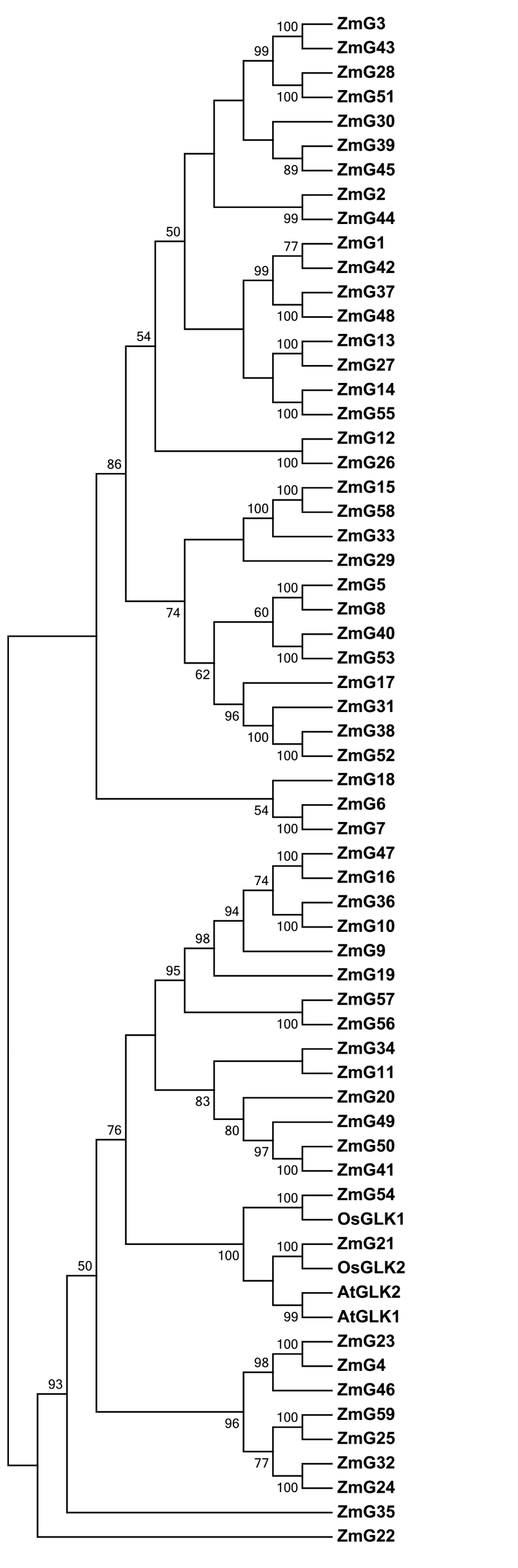

Supplement: S2 Fig — (TIF) [file pone.0161763.s002.tif]

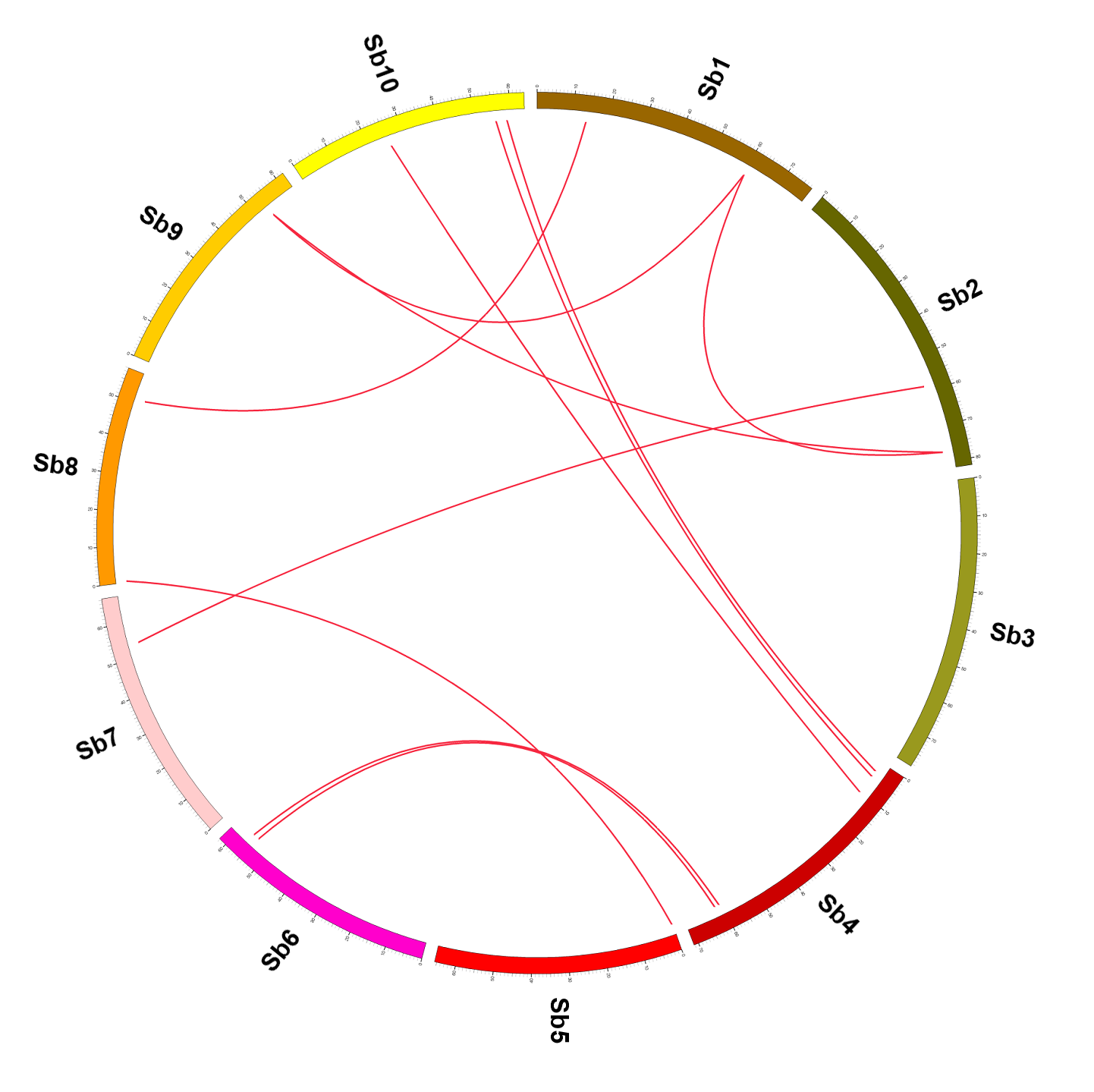

Supplement: S3 Fig — Numbers along each chromosome box indicate sequence lengths in megabases. All the syntenic genes were located in sorghum chromosome, and linked by red lines. (TIF) [file pone.0161763.s003.tif]

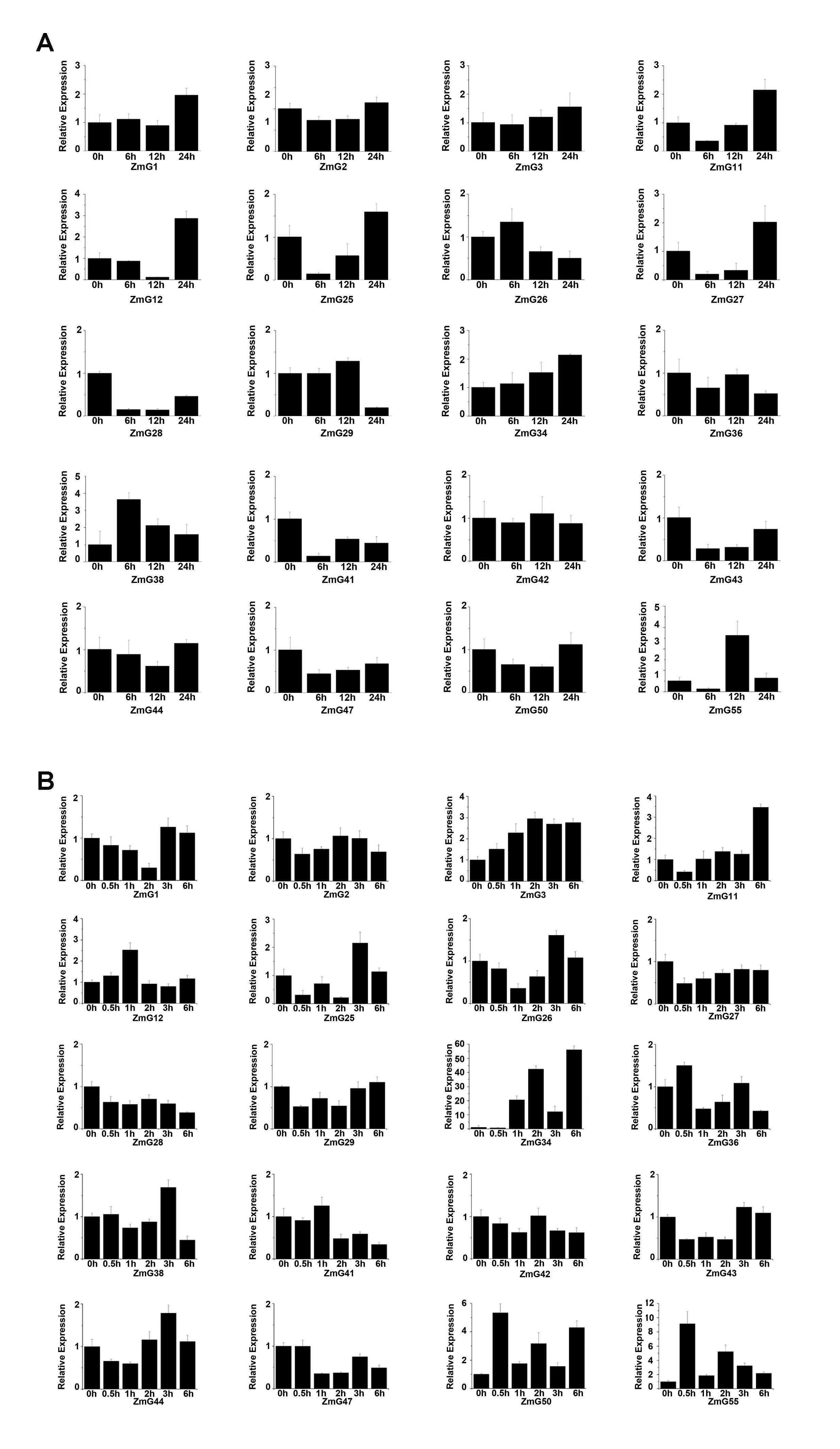

Supplement: S4 Fig — The Y-axis is the scale of relative expression levels. The X-axis is time courses of stress treatments. Error bars, +SE. (A) Relative expression levels of the twenty stress-responsive G2-like genes in responsive to drought stress. Seedlings were sampled at 0 h (CK), 6 h, 12 h, and 24 h after drought treatment. (B) Relative expression levels of the twenty stress- responsive G2-like genes in response to low temperature treatment (4°C). Seedlings were sampled at 0 h (CK), 0.5 h, 1 h, 2 h, 3 h and 6 h after 4°C treatment. (TIF) [file pone.0161763.s004.tif]
