# Supplementary material for: Molecular Evolution and Genetic Variation of G2-Like Transcription Factor Genes in Maize
Source: PLoS One. 2016 Aug 25;11(8):e0161763. doi: 10.1371/journal.pone.0161763 (PMC4999087; doi:10.1371/journal.pone.0161763)
Supplement: S1 Table — (DOC) [file pone.0161763.s005.doc]

S1 Table Basic information about *G2-like* genes in maize

| Gene name | Translation | pI / MW (Da) | Size (aa) | Chr. | Exon number | Subcellular localization prediction |  |
| --- | --- | --- | --- | --- | --- | --- | --- |
| *ZmG1* | GRMZM2G009060_P01 | 7.28 / 39861.23 | 359 | 1 | 6 | N |  |
| *ZmG2* | GRMZM2G006477_P01 | 6.16 / 49645.57 | 450 | 1 | 7 | N |  |
| *ZmG3* | GRMZM2G113742_P01 | 9.43 / 29156.18 | 269 | 1 | 6 | N |  |
| *ZmG4* | GRMZM2G056400_P01 | 8.82 / 47557.66 | 458 | 1 | 6 | N |  |
| *ZmG5* | GRMZM5G887276_P03 | 8.73 / 46203.21 | 434 | 1 | 6 | N |  |
| *ZmG6* | GRMZM2G039074_P02 | 9.80 / 45382.56 | 427 | 1 | 6 | N |  |
| *ZmG7* | GRMZM2G034563_P01 | 6.92 / 26111.26 | 248 | 1 | 4 | E |  |
| *ZmG8* | AC234520.1_FGP003 | 9.56 / 19591.43 | 186 | 1 | 3 | N |  |
| *ZmG9* | GRMZM2G124540_P01 | 6.12 / 35804.38 | 338 | 1 | 5 | N |  |
| *ZmG10* | GRMZM2G379656_P01 | 5.15 / 16687.42 | 145 | 1 | 3 | N |  |
| *ZmG11* | GRMZM2G117193_P03 | 7.22 / 53169.28 | 495 | 1 | 1 | N |  |
| *ZmG12* | GRMZM2G125704_P02 | 9.19 / 30274.45 | 271 | 2 | 6 | N |  |
| *ZmG13* | GRMZM2G035370_P01 | 7.80 / 51625.62 | 467 | 2 | 7 | N |  |
| *ZmG14* | GRMZM2G069525_P01 | 9.08 / 33135.25 | 312 | 2 | 6 | N/C/M |  |
| *ZmG15* | GRMZM2G173882_P01 | 7.02 / 36735.53 | 345 | 2 | 5 | N |  |
| *ZmG16* | GRMZM5G846506_P01 | 9.47 / 41247.17 | 381 | 2 | 5 | N |  |
| *ZmG17* | GRMZM2G370425_P01 | 6.65 / 43773.75 | 409 | 3 | 5 | N |  |
| *ZmG18* | GRMZM2G348238_P01 | 5.80 / 52172.00 | 499 | 3 | 4 | N |  |
| *ZmG19* | GRMZM2G074908_P01 | 5.68 / 32180.44 | 304 | 3 | 2 | N |  |
| *ZmG20* | GRMZM2G087804_P03 | 6.72 / 49254.78 | 461 | 3 | 5 | N |  |
| *ZmG21* | GRMZM2G396825_P01 | 8.54 / 32555.42 | 297 | 3 | 5 | C |  |
| *ZmG22* | GRMZM2G175827_P02 | 9.16 / 49074.33 | 468 | 4 | 6 | N |  |
| *ZmG23* | GRMZM2G070865_P01 | 6.71 / 39524.16 | 372 | 4 | 6 | N |  |
| *ZmG24* | GRMZM2G315506_P01 | 8.66 / 37640.56 | 357 | 4 | 7 | N |  |
| *ZmG25* | GRMZM2G168002_P04 | 6.59 / 28660.53 | 270 | 4 | 4 | N |  |
| *ZmG26* | AC233960.1_FGP003 | 8.40 / 30635.80 | 279 | 5 | 6 | N |  |
| *ZmG27* | GRMZM2G052544_P01 | 7.22 / 40783.49 | 376 | 5 | 6 | N |  |
| *ZmG28* | GRMZM2G083472_P01 | 5.69 / 33179.87 | 309 | 5 | 3 | N |  |
| *ZmG29* | GRMZM2G010920_P01 | 6.61 / 48305.91 | 438 | 5 | 7 | N |  |
| *ZmG30* | GRMZM2G477238_P01 | 6.21 / 58738.69 | 540 | 5 | 6 | N |  |
| *ZmG31* | GRMZM2G060834_P01 | 7.41 / 40092.95 | 380 | 5 | 6 | N |  |
| *ZmG32* | GRMZM2G106185_P02 | 10.17/34599.99 | 378 | 5 | 5 | N |  |
| *ZmG33* | GRMZM2G060485_P01 | 6.36 / 58845.41 | 545 | 5 | 5 | N |  |
| *ZmG34* | GRMZM2G379167_P01 | 6.57 / 44260.81 | 402 | 5 | 6 | N |  |
| *ZmG35* | GRMZM2G171468_P01 | 5.33 / 38386.96 | 362 | 5 | 5 | N |  |
| *ZmG36* | GRMZM2G701218_P01 | 6.53 / 46434.86 | 414 | 6 | 6 | N |  |
| *ZmG37* | AC219020.4_FGP002 | 6.17 / 59226.91 | 554 | 6 | 6 | N |  |
| *ZmG38* | GRMZM2G117854_P01 | 6.08 / 28416.91 | 257 | 6 | 6 | N |  |
| *ZmG39* | GRMZM2G100709_P01 | 7.71 / 28085.27 | 256 | 6 | 5 | N |  |
| *ZmG40* | GRMZM2G398055_P01 | 7.09 / 31420.01 | 312 | 6 | 1 | N |  |
| *ZmG41* | AC155434.2_FGP005 | 7.78 / 39792.34 | 367 | 7 | 6 | N |  |
| *ZmG42* | GRMZM2G064197_P01 | 8.48 / 27732.51 | 256 | 7 | 6 | N |  |
| *ZmG43* | GRMZM2G162409_P05 | 4.62 / 30102.02 | 441 | 7 | 8 | N |  |
| *ZmG44* | GRMZM2G173943_P02 | 5.95 / 34026.39 | 312 | 7 | 6 | N |  |
| *ZmG45* | GRMZM2G082264_P01 | 6.98 / 48352.37 | 477 | 7 | 6 | N |  |
| *ZmG46* | GRMZM2G100176_P01 | 7.06 / 39865.83 | 369 | 7 | 5 | N |  |
| *ZmG47* | AC234155.1_FGP002 | 6.23 / 44110.34 | 393 | 8 | 6 | N |  |
| *ZmG48* | GRMZM2G067702_P01 | 6.55 / 23717.77 | 219 | 8 | 1 | N |  |
| *ZmG49* | GRMZM2G471600_P01 | 6.83 / 30828.47 | 299 | 8 | 1 | C |  |
| *ZmG50* | GRMZM2G081671_P01 | 7.70 / 37367.17 | 343 | 9 | 6 | N |  |
| *ZmG51* | GRMZM2G333083_P01 | 6.48 / 56361.35 | 521 | 9 | 1 | N |  |
| *ZmG52* | GRMZM2G454449_P01 | 8.59 / 28049.28 | 253 | 9 | 5 | N |  |
| *ZmG53* | GRMZM2G026833_P01 | 5.20 / 50523.81 | 476 | 9 | 6 | N |  |
| *ZmG54* | GRMZM2G374986_P01 | 9.30 / 42557.70 | 391 | 1 | 3 | N |  |
| *ZmG55* | GRMZM2G124495_P01 | 8.15 / 49168.55 | 458 | 10 | 7 | M |  |
| *ZmG56* | GRMZM2G016370_P01 | 6.08 / 48702.17 | 453 | 10 | 4 | N |  |
| *ZmG57* | GRMZM2G159119_P01 | 6.58 / 49338.97 | 459 | 10 | 4 | N |  |
| *ZmG58* | GRMZM2G090230_P01 | 8.74 / 35491.89 | 328 | 10 | 6 | N |  |
| *ZmG59* | GRMZM2G123308_P02 | 7.35 / 37355.24 | 356 | 10 | 1 | N |  |

C: Chloroplast; M: Mitochondria; E: Extracellular; N: Nuclear
