# Supplementary material for: Molecular Evolution and Genetic Variation of G2-Like Transcription Factor Genes in Maize
Source: PLoS One. 2016 Aug 25;11(8):e0161763. doi: 10.1371/journal.pone.0161763 (PMC4999087; doi:10.1371/journal.pone.0161763)
Supplement: S2 Table — (DOCX) [file pone.0161763.s006.docx]

S2 Table Motif sequences identified using MEME tools

| Motif No. | Multilevel consensus sequence | Motif Name |
| --- | --- | --- |
| 1 | KATPKTILELMNVKGLTIYHVKSHLQKYR | MYB-DBD |
| 2 | YNTDKKPRMRWTPELHRRFVHAVNQLGGH | MYB-DBD |
| 3 | MQIEVQRRLHEQLEVQRHLQLRIEAQGKY | MYB-CC-LHEQLE |
| 4 | FRDCIRRLEEERRKIEVFKRELPLCLRLLADVIDEMKEEMDKYV | Terpene-synth-C |
| 5 | METFPAHPDLSLHISPPSPA |  |
| 6 | DKPKWMSTAQLWTNDCQKSDEPPKKQDKEICSCEPVKLNAC |  |
| 7 | MQSILEKAQETLAKHNCGSA | SAB |
| 8 | MGLDVGEIGMGLDLGLDLRHFAAKAVGGM |  |
| 9 | FENTCSANKLDLNTHNVDDTNQAYRHFDLNGFNW |  |
| 10 | PVAQQIVVVGGIWVPPEQ |  |
| 11 | LSKNIQAQANASTSKNAIGCTPIADRIPGTNAATMSSTNVVPQAEKTIQI |  |
| 12 | FCRKRPHEHETQFALNRSLSERRMAHLQNEEGYHKAEFGYESDTEIVHEY |  |
| 13 | AAMARPIRGIPVYNHPH |  |
| 14 | CPPNLEFTLGRPIWQ |  |
| 15 | WEQGLPRPDELMPLTQPLIPPHLAVAFDI |  |
| 16 | CFSNAITDVKESSSVHRLEPIQIEFVESSTNSYLSVAEGFI |  |
| 17 | REDWTGFPSESNTGRMSMHSRSLKDQTMQSKSLEILSDMNSSCVSETTSC |  |
| 18 | FQPFEKEKKKKDKEEKQRAELELPLPAAASSAVVGDSCDRAGATDTDTDT | ABA-WDS |
| 19 | HHHHHHHL |  |
| 20 | CFCEPCHVAAGAWTTAGCGFGTRVVGGFP |  |
