# Supplementary material for: Molecular Evolution and Genetic Variation of G2-Like Transcription Factor Genes in Maize
Source: PLoS One. 2016 Aug 25;11(8):e0161763. doi: 10.1371/journal.pone.0161763 (PMC4999087; doi:10.1371/journal.pone.0161763)
Supplement: S3 Table — (DOCX) [file pone.0161763.s007.docx]

S3 Table *G2-like* genes in Sorghum

| Gene Name | Gene ID | Gene Name | Gene ID |
| --- | --- | --- | --- |
| SbG1 | Sb01g036680.1 | SbG24 | Sb08g019940.1 |
| SbG2 | Sb02g043320.1 | SbG25 | Sb05g000480.1 |
| SbG3 | Sb09g023830.1 | SbG26 | Sb01g014354.1 |
| SbG4 | Sb10g026550.1 | SbG27 | Sb09g024090.1 |
| SbG5 | Sb04g030830.1 | SbG28 | Sb07g004100.1 |
| SbG6 | Sb06g027405.1 | SbG29 | Sb04g032130.1 |
| SbG7 | Sb04g004930.1 | SbG30 | Sb04g036955.1 |
| SbG8 | Sb01g036440.1 | SbG31 | Sb08g019720.1 |
| SbG9 | Sb02g010520.1 | SbG32 | Sb03g004090.1 |
| SbG10 | Sb10g029200.1 | SbG33 | Sb03g047330.1 |
| SbG11 | Sb04g003140.1 | SbG34 | Sb01g007130.1 |
| SbG12 | Sb02g020700.1 | SbG35 | Sb06g025600.1 |
| SbG13 | Sb10g021360.1 | SbG36 | Sb02g001600.1 |
| SbG14 | Sb06g031970.1 | SbG37 | Sb09g020340.1 |
| SbG15 | Sb04g008670.1 | SbG38 | Sb03g039610.1 |
| SbG16 | Sb07g020820.1 | SbG39 | Sb10g008400.1 |
| SbG17 | Sb04g031030.1 | SbG40 | Sb03g000400.1 |
| SbG18 | Sb01g007030.1 | SbG41 | Sb10g025500.1 |
| SbG19 | Sb08g003180.1 | SbG42 | Sb01g013080.1 |
| SbG20 | Sb07g021290.1 | SbG43 | Sb03g012625.1 |
| SbG21 | Sb10g026930.1 | SbG44 | Sb02g024110.1 |
| SbG22 | Sb08g000510.1 | SbG45 | Sb02g020690.1 |
| SbG23 | Sb01g048420.1 |  |  |
