# Supplementary material for: Molecular Evolution and Genetic Variation of G2-Like Transcription Factor Genes in Maize
Source: PLoS One. 2016 Aug 25;11(8):e0161763. doi: 10.1371/journal.pone.0161763 (PMC4999087; doi:10.1371/journal.pone.0161763)
Supplement: S4 Table — (DOCX) [file pone.0161763.s008.docx]

S4 Table Collinear gene pairs in sorghum

| Gene name | Chr. Location | Gene position | Gene name | Chr. Location | Gene position |
| --- | --- | --- | --- | --- | --- |
| Sb10g026550.1 | sb10 | 55969655-55970188 | Sb04g004930.1 | sb4 | 4724420-4726981 |
| Sb10g021360.1 | sb10 | 47244434-47246577 | Sb04g008670.1 | sb4 | 10179814-10181981 |
| Sb02g043320.1 | sb2 | 77131486-77134498 | Sb01g036680.1 | sb1 | 60305125-60308495 |
| Sb04g032130.1 | sb4 | 62134817-62137573 | Sb06g025600.1 | sb6 | 54597438-54600310 |
| Sb04g003140.1 | sb4 | 2942965-2945111 | Sb10g029200.1 | sb10 | 59035792-59037297 |
| Sb06g027405.1 | sb6 | 56303673-56305290 | Sb04g030830.1 | sb4 | 60829092-60831886 |
| Sb07g020820.1 | sb7 | 53875498-53880521 | Sb02g024110.1 | sb2 | 58128757-58131105 |
| Sb08g019940.1 | sb8 | 50931974-50934229 | Sb01g014354.1 | sb1 | 13622707-13624434 |
| Sb08g000510.1 | sb8 | 443187-444804 | Sb05g000480.1 | sb5 | 330603-332440 |
| Sb09g023830.1 | sb9 | 53471303-53474209 | Sb01g036680.1 | sb1 | 60305125-60308495 |
| Sb09g023830.1 | sb9 | 53471303-53474209 | Sb02g043320.1 | sb2 | 77131486-77134498 |
