# Supplementary material for: Molecular Evolution and Genetic Variation of G2-Like Transcription Factor Genes in Maize
Source: PLoS One. 2016 Aug 25;11(8):e0161763. doi: 10.1371/journal.pone.0161763 (PMC4999087; doi:10.1371/journal.pone.0161763)
Supplement: S5 Table — (DOCX) [file pone.0161763.s009.docx]

S5 Table Orthologous gene pairs between maize and sorghum

| Translation | Chr. Location | Gene position | Gene name | Chr. Location | Gene position |
| --- | --- | --- | --- | --- | --- |
| GRMZM2G031870_P01 | zm1 | 280750442-280751223 | Sb01g007030.1 | sb1 | 5945813-5952018 |
| GRMZM2G124540_P01 | zm1 | 280218237-280220278 | Sb01g007130.1 | sb1 | 6054669-6056711 |
| GRMZM2G034563_P01 | zm1 | 165135132-165137087 | Sb01g014354.1 | sb1 | 13622707-13624434 |
| GRMZM2G379656_P01 | zm1 | 234417882-234419889 | Sb01g019980.1 | sb1 | 21921315-21925396 |
| GRMZM2G006477_P01 | zm1 | 54343607-54350368 | Sb01g036440.1 | sb1 | 60049356-60054379 |
| GRMZM2G009060_P01 | zm1 | 52394843-52397225 | Sb01g036680.1 | sb1 | 60305125-60308495 |
| GRMZM2G056400_P01 | zm1 | 215606266-215611664 | Sb07g020820.1 | sb7 | 53875498-53880521 |
| AC234520.1_FGP003 | zm1 | 164369081-164369911 | Sb08g019720.1 | sb8 | 50726046-50735826 |
| GRMZM2G034563_P01 | zm1 | 165135132-165137087 | Sb08g019940.1 | sb8 | 50931974-50934229 |
| GRMZM2G090230_P01 | zm10 | 137353841-137357660 | Sb06g025600.1 | sb6 | 54597438-54600310 |
| GRMZM2G124495_P01 | zm10 | 147140446-147143263 | Sb06g031970.1 | sb6 | 60252217-60254843 |
| GRMZM2G123308_P01 | zm10 | 77334656-77336671 | Sb07g004100.1 | sb7 | 5154354-5158043 |
| GRMZM2G016370_P01 | zm10 | 5384582-5386647 | Sb08g003180.1 | sb8 | 3527574-3529715 |
| GRMZM2G159119_P01 | zm10 | 5122182-5124287 | Sb08g003180.1 | sb8 | 3527574-3529715 |
| GRMZM2G173882_P01 | zm2 | 236187625-236189534 | Sb01g007130.1 | sb1 | 6054669-6056711 |
| GRMZM2G173882_P01 | zm2 | 236187625-236189534 | Sb02g001600.1 | sb2 | 1460607-1462435 |
| GRMZM2G090061_P01 | zm2 | 173424177-173426267 | Sb02g020690.1 | sb2 | 49999856-50005174 |
| GRMZM2G125704_P01 | zm2 | 12347435-12350429 | Sb04g030830.1 | sb4 | 60829092-60831886 |
| GRMZM2G069525_P01 | zm2 | 16870336-16874086 | Sb04g032130.1 | sb4 | 62134817-62137573 |
| GRMZM5G846506_P01 | zm2 | 153095610-153097560 | Sb05g000480.1 | sb5 | 330603-332440 |
| GRMZM2G069525_P01 | zm2 | 16870336-16874086 | Sb06g025600.1 | sb6 | 54597438-54600310 |
| GRMZM2G125704_P01 | zm2 | 12347435-12350429 | Sb06g027405.1 | sb6 | 56303673-56305290 |
| GRMZM2G035370_P01 | zm2 | 3105142-3108209 | Sb06g031970.1 | sb6 | 60252217-60254843 |
| GRMZM5G846506_P01 | zm2 | 153095610-153097560 | Sb08g000510.1 | sb8 | 443187-444804 |
| GRMZM2G370425_P01 | zm3 | 114430011-114433164 | Sb01g014354.1 | sb1 | 13622707-13624434 |
| GRMZM2G087804_P01 | zm3 | 1464399-1469199 | Sb03g000400.1 | sb3 | 290626-298022 |
| GRMZM2G348238_P01 | zm3 | 10521217-10523452 | Sb03g004090.1 | sb3 | 4346545-4348435 |
| GRMZM2G396825_P01 | zm3 | 55167963-55170910 | Sb03g012625.1 | sb3 | 15089017-15090349 |
| GRMZM2G074908_P01 | zm3 | 180256139-180257888 | Sb03g039610.1 | sb3 | 67284776-67287499 |
| GRMZM2G370425_P01 | zm3 | 114430011-114433164 | Sb08g019940.1 | sb8 | 50931974-50934229 |
| GRMZM2G070865_P01 | zm4 | 158068289-158073370 | Sb04g031030.1 | sb4 | 61020712-61025629 |
| GRMZM2G315506_P01 | zm4 | 31306072-31309853 | Sb07g004100.1 | sb7 | 5154354-5158043 |
| GRMZM2G175827_P01 | zm4 | 80073206-80076285 | Sb07g020820.1 | sb7 | 53875498-53880521 |
| GRMZM2G168002_P01 | zm4 | 83196578-83198800 | Sb07g021290.1 | sb7 | 55255162-55256775 |
| GRMZM2G171468_P01 | zm5 | 6140225-6142369 | Sb01g007130.1 | sb1 | 6054669-6056711 |
| GRMZM2G065194_P01 | zm5 | 15650410-15655937 | Sb01g013080.1 | sb1 | 12109925-12117727 |
| GRMZM2G010920_P01 | zm5 | 77748895-77751969 | Sb04g003140.1 | sb4 | 2942965-2945111 |
| GRMZM2G083472_P01 | zm5 | 58136739-58139375 | Sb04g003140.1 | sb4 | 2942965-2945111 |
| GRMZM2G052544_P01 | zm5 | 86260279-86263490 | Sb04g004930.1 | sb4 | 4724420-4726981 |
| GRMZM2G060485_P01 | zm5 | 93237316-93240361 | Sb04g005580.1 | sb4 | 5463889-5468190 |
| GRMZM2G477238_P01 | zm5 | 145105147-145107376 | Sb04g008670.1 | sb4 | 10179814-10181981 |
| AC233960.1_FGP003 | zm5 | 199978948-199981420 | Sb04g030830.1 | sb4 | 60829092-60831886 |
| GRMZM2G060834_P01 | zm5 | 199288141-199293157 | Sb04g031030.1 | sb4 | 61020712-61025629 |
| GRMZM2G106185_P01 | zm5 | 194250832-194253964 | Sb04g032130.1 | sb4 | 62134817-62137573 |
| GRMZM2G379167_P01 | zm5 | 215472451-215474846 | Sb04g036955.1 | sb4 | 66716431-66718597 |
| AC233960.1_FGP003 | zm5 | 199978948-199981420 | Sb06g027405.1 | sb6 | 56303673-56305290 |
| GRMZM2G477238_P01 | zm5 | 145105147-145107376 | Sb10g021360.1 | sb10 | 47244434-47246577 |
| GRMZM2G052544_P01 | zm5 | 86260279-86263490 | Sb10g026550.1 | sb10 | 55969655-55970188 |
| GRMZM2G010920_P01 | zm5 | 77748895-77751969 | Sb10g029200.1 | sb10 | 59035792-59037297 |
| GRMZM2G083472_P01 | zm5 | 58136739-58139375 | Sb10g029200.1 | sb10 | 59035792-59037297 |
| GRMZM2G701218_P01 | zm6 | 155178001-155181206 | Sb01g036680.1 | sb1 | 60305125-60308495 |
| GRMZM2G398055_P01 | zm6 | 147024173-147026212 | Sb09g020340.1 | sb9 | 49616534-49617892 |
| GRMZM2G701218_P01 | zm6 | 155178001-155181206 | Sb09g023830.1 | sb9 | 53471303-53474209 |
| GRMZM2G117854_P01 | zm6 | 155520554-155524228 | Sb09g024090.1 | sb9 | 53647365-53650935 |
| AC219020.4_FGP002 | zm6 | 105751774-105754070 | Sb10g021360.1 | sb10 | 47244434-47246577 |
| GRMZM2G100709_P01 | zm6 | 93229968-93238170 | Sb10g026930.1 | sb10 | 56337896-56341893 |
| AC155434.2_FGP005 | zm7 | 173817875-173820052 | Sb01g036680.1 | sb1 | 60305125-60308495 |
| GRMZM2G100176_P01 | zm7 | 2629130-2631159 | Sb02g001600.1 | sb2 | 1460607-1462435 |
| GRMZM2G162409_P01 | zm7 | 39503887-39510076 | Sb02g010520.1 | sb2 | 16094071-16100737 |
| GRMZM2G064197_P01 | zm7 | 83143687-83149297 | Sb02g020690.1 | sb2 | 49999856-50005174 |
| GRMZM2G173943_P01 | zm7 | 83267783-83272193 | Sb02g020700.1 | sb2 | 50012303-50017233 |
| GRMZM2G082264_P01 | zm7 | 107233099-107239626 | Sb02g024110.1 | sb2 | 58128757-58131105 |
| AC155434.2_FGP005 | zm7 | 173817875-173820052 | Sb02g043320.1 | sb2 | 77131486-77134498 |
| GRMZM2G082264_P01 | zm7 | 107233099-107239626 | Sb07g020820.1 | sb7 | 53875498-53880521 |
| AC234155.1_FGP002 | zm8 | 119581337-119584279 | Sb01g036680.1 | sb1 | 60305125-60308495 |
| GRMZM2G067702_P01 | zm8 | 156720384-156721759 | Sb03g047330.1 | sb3 | 74205803-74207284 |
| GRMZM2G471600_P01 | zm8 | 104819411-104820748 | Sb09g020340.1 | sb9 | 49616534-49617892 |
| AC234155.1_FGP002 | zm8 | 119581337-119584279 | Sb09g023830.1 | sb9 | 53471303-53474209 |
| GRMZM2G374986_P01 | zm9 | 153449284-153453177 | Sb01g048420.1 | sb1 | 71443983-71448752 |
| GRMZM2G081671_P01 | zm9 | 109611246-109613880 | Sb04g004930.1 | sb4 | 4724420-4726981 |
| GRMZM2G333083_P01 | zm9 | 60952018-60954746 | Sb04g008670.1 | sb4 | 10179814-10181981 |
| GRMZM2G026833_P01 | zm9 | 2711381-2713815 | Sb10g008400.1 | sb10 | 8592581-8595107 |
| GRMZM2G333083_P01 | zm9 | 60952018-60954746 | Sb10g021360.1 | sb10 | 47244434-47246577 |
| GRMZM2G360523_P01 | zm9 | 100983990-100992750 | Sb10g025500.1 | sb10 | 54822361-54824558 |
| GRMZM2G081671_P01 | zm9 | 109611246-109613880 | Sb10g026550.1 | sb10 | 55969655-55970188 |
