# Supplementary material for: Molecular Evolution and Genetic Variation of G2-Like Transcription Factor Genes in Maize
Source: PLoS One. 2016 Aug 25;11(8):e0161763. doi: 10.1371/journal.pone.0161763 (PMC4999087; doi:10.1371/journal.pone.0161763)
Supplement: S6 Table — (DOCX) [file pone.0161763.s010.docx]

S6 Table Genetic diversity of *G2-like* genes between tropical (Tro) and temperate (Tem) inbred maize lines

| Name | Gene identifier | Type | Nucleotide Diversity (%) | | | Haplotype diversity | | Genetic Differentiation | | | | | |
| --- | --- | --- | --- | --- | --- | --- | --- | --- | --- | --- | --- | --- | --- |
|  |  |  | Tro | Tem | Dxy | Tro | Tem | Gst | Nst | Fst | χ^2^ | df | P |
| *ZmG1* | GRMZM2G009060_T01 | intermediate | 0.00133 | 0.0012 | 0.00124 | 0.62145 | 0.58859 | -0.0042 | -0.01955 | -0.01954 | 6.977 | 6 | 0.323 |
| *ZmG2* | GRMZM2G006477_T01 | intermediate | 0.00059 | 0.00071 | 0.00067 | 0.47252 | 0.57658 | 0.00467 | 0.0332 | 0.03317 | 6.072 | 4 | 0.1938 |
| *ZmG3* | GRMZM2G113742_T01 | intermediate | 0.0012 | 0.0004 | 0.00082 | 0.42642 | 0.10511 | 0.0338 | 0.02236 | 0.02247 | 7.381 | 4 | 0.1171 |
| *ZmG4* | GRMZM2G056400_T01 | intermediate | 0.0008 | 0.00056 | 0.00074 | 0.68528 | 0.54354 | 0.03316 | 0.08256 | 0.08255 | 8.192 | 4 | 0.0848 |
| *ZmG5* | GRMZM5G887276_T03 | diversity | 0.0018 | 0.00192 | 0.00213 | 0.90957 | 0.85736 | 0.02246 | 0.12787 | 0.12774 | 48.435 | 18 | 0.0001 *** |
| *ZmG6* | GRMZM2G039074_T02 | diversity | 0.00155 | 0.00129 | 0.0014 | 0.79344 | 0.72523 | 0.00035 | -0.01247 | -0.01245 | 12.552 | 10 | 0.2498 |
| *ZmG7* | GRMZM2G034563_T01 | intermediate | 0.00006 | 0.00021 | 0.00013 | 0.04167 | 0.15315 | 0.0063 | 0.01143 | 0.01143 | 1.691 | 1 | 0.1934 |
| *ZmG8* | AC234520.1_FGT003 | conserved | 0 | 0 | 0 | 0 | 0 | - | - | - | - | - | - |
| *ZmG9* | GRMZM2G124540_T01 | diversity | 0.0029 | 0.00239 | 0.00295 | 0.91046 | 0.81832 | 0.01764 | 0.10367 | 0.10353 | 25.644 | 16 | 0.0592 |
| *ZmG10* | GRMZM2G379656_T01 | conserved | 0 | 0 | 0 | 0 | 0 | - | - | - | - | - | - |
| *ZmG11* | GRMZM2G117193_T03 | diversity | 0.00106 | 0.00094 | 0.00119 | 0.86791 | 0.60511 | 0.09493 | 0.15642 | 0.15644 | 36.468 | 9 | 0*** |
| *ZmG12* | GRMZM2G125704_T02 | intermediate | 0.00083 | 0.00013 | 0.0005 | 0.51596 | 0.10511 | 0.04825 | 0.03559 | 0.03571 | 10.477 | 6 | 0.1059 |
| *ZmG14* | GRMZM2G069525_T01 | diversity | 0.00164 | 0.00185 | 0.00204 | 0.73582 | 0.76276 | 0.02025 | 0.14274 | 0.14257 | 23.977 | 10 | 0.0077** |
| *ZmG15* | GRMZM2G173882_T01 | diversity | 0.00189 | 0.00184 | 0.00208 | 0.85372 | 0.8018 | 0.03735 | 0.10233 | 0.1023 | 28.724 | 12 | 0.0043 ** |
| *ZmG16* | GRMZM5G846506_T01 | intermediate | 0.00023 | 0 | 0.00013 | 0.25443 | 0 | 0.06006 | 0.12766 | 0.12766 | 5.88 | 1 | 0.0153 * |
| *ZmG17* | GRMZM2G370425_T01 | diversity | 0.002 | 0.0023 | 0.00245 | 0.74468 | 0.67417 | 0.04537 | 0.12142 | 0.12142 | 23.197 | 8 | 0.0031 ** |
| *ZmG18* | GRMZM2G348238_T01 | intermediate | 0.00059 | 0.00046 | 0.00053 | 0.6578 | 0.58559 | 0.0041 | 0.01136 | 0.01136 | 6.757 | 6 | 0.3439 |
| *ZmG19* | GRMZM2G074908_T01 | intermediate | 0.00009 | 0.00023 | 0.00016 | 0.08156 | 0.2042 | 0.00069 | -0.00424 | -0.00423 | 2.762 | 2 | 0.2513 |
| *ZmG20* | GRMZM2G087804_T03 | intermediate | 0.00065 | 0.00023 | 0.00047 | 0.62411 | 0.2958 | 0.03683 | 0.07153 | 0.07155 | 12.188 | 6 | 0.0579 |
| *ZmG22* | GRMZM2G175827_T02 | intermediate | 0.0003 | 0.00025 | 0.00045 | 0.42199 | 0.34835 | 0.22724 | 0.377 | 0.377 | 20.243 | 1 | 0.0000 *** |
| *ZmG23* | GRMZM2G070865_T01 | intermediate | 0.00022 | 0.00019 | 0.00021 | 0.1977 | 0.15616 | -0.0017 | 0.00238 | 0.00238 | 5.724 | 4 | 0.2207 |
| *ZmG24* | GRMZM2G315506_T01 | diversity | 0.00132 | 0.00071 | 0.00108 | 0.71986 | 0.56907 | 0.0166 | 0.06298 | 0.06297 | 18.248 | 9 | 0.0324 * |
| *ZmG25* | GRMZM2G168002_T04 | conserved | 0 | 0 | 0 | 0 | 0 | - | - | - | - | - | - |
| *ZmG26* | AC233960.1_FGT003 | diversity | 0.00124 | 0.00107 | 0.00117 | 0.57624 | 0.63664 | 0.03818 | 0.01452 | 0.01463 | 10.887 | 7 | 0.1436 |
| *ZmG27* | GRMZM2G052544_T01 | diversity | 0.0023 | 0.00198 | 0.00214 | 0.88298 | 0.84835 | 0.00546 | -0.00154 | -0.00151 | 24.726 | 17 | 0.101 |
| *ZmG29* | GRMZM2G010920_T01 | intermediate | 0.00083 | 0.0002 | 0.00054 | 0.30496 | 0.24625 | 0.00662 | 0.04257 | 0.04259 | 12.678 | 7 | 0.0804 |
| *ZmG30* | GRMZM2G477238_T01 | diversity | 0.00595 | 0.00345 | 0.00528 | 0.81915 | 0.85285 | 0.0355 | 0.11046 | 0.11025 | 37.191 | 17 | 0.0032 ** |
| *ZmG31* | GRMZM2G060834_T01 | intermediate | 0.00079 | 0.00087 | 0.00082 | 0.3617 | 0.34084 | -0.0076 | -0.0172 | -0.01719 | 3.239 | 4 | 0.5187 |
| *ZmG32* | GRMZM2G106185_T02 | intermediate | 0.00026 | 0.00053 | 0.00053 | 0.25443 | 0.51351 | 0.1441 | 0.24648 | 0.24648 | 13.304 | 1 | 0.0003*** |
| *ZmG33* | GRMZM2G060485_T01 | diversity | 0.00122 | 0.00089 | 0.00105 | 0.76241 | 0.65766 | -0.0006 | -0.00574 | -0.00574 | 8.981 | 10 | 0.5339 |
| *ZmG34* | GRMZM2G379167_T01 | diversity | 0.00412 | 0.00565 | 0.00513 | 0.81915 | 0.78679 | 0.03373 | 0.04692 | 0.04716 | 16.751 | 10 | 0.0801 |
| *ZmG35* | GRMZM2G171468_T01 | diversity | 0.00138 | 0.00069 | 0.00115 | 0.77305 | 0.49099 | 0.04783 | 0.0969 | 0.09692 | 16.628 | 8 | 0.0342 * |
| *ZmG36* | GRMZM2G701218_T01 | intermediate | 0.00036 | 0 | 0.00019 | 0.19681 | 0 | 0.02505 | 0.03478 | 0.03482 | 4.095 | 3 | 0.2514 |
| *ZmG37* | AC219020.4_FGT002 | diversity | 0.00222 | 0.00155 | 0.00221 | 0.86791 | 0.74775 | 0.05724 | 0.1483 | 0.14823 | 41.54 | 17 | 0.0008*** |
| *ZmG38* | GRMZM2G117854_T01 | diversity | 0.00189 | 0.00139 | 0.00162 | 0.66667 | 0.58408 | 0.00122 | -0.01115 | -0.01108 | 9.36 | 7 | 0.2278 |
| *ZmG39* | GRMZM2G100709_T01 | diversity | 0.00136 | 0.00142 | 0.00154 | 0.69149 | 0.59206 | 0.12115 | 0.09497 | 0.09509 | 31.042 | 5 | 0*** |
| *ZmG41* | AC155434.2_FGT005 | diversity | 0.00141 | 0.00188 | 0.00173 | 0.7633 | 0.79129 | 0.00359 | 0.04759 | 0.04751 | 22.052 | 14 | 0.0775 |
| *ZmG43* | GRMZM2G162409_T05 | conserved | 0.00005 | 0 | 0.00003 | 0.04167 | 0 | 0.0002 | 0 | 0 | 0.78 | 1 | 0.3771 |
| *ZmG45* | GRMZM2G082264_T01 | intermediate | 0.00062 | 0.00029 | 0.00048 | 0.48316 | 0.20571 | 0.02644 | 0.04525 | 0.04524 | 10.772 | 5 | 0.0561 |
| *ZmG46* | GRMZM2G100176_T01 | diversity | 0.00181 | 0.00203 | 0.00194 | 0.87057 | 0.8994 | 0.00836 | 0.01359 | 0.01361 | 27.242 | 18 | 0.0746 |
| *ZmG47* | AC234155.1_FGT002 | intermediate | 0.00124 | 0.00037 | 0.00096 | 0.44592 | 0.42943 | 0.04056 | 0.16633 | 0.16619 | 17.427 | 4 | 0.0016** |
| *ZmG48* | GRMZM2G067702_T01 | intermediate | 0.0012 | 0.00059 | 0.00092 | 0.54433 | 0.32432 | 0.00976 | 0.02115 | 0.02116 | 4.942 | 3 | 0.1761 |
| *ZmG49* | GRMZM2G471600_T01 | conserved | 0 | 0 | 0 | 0 | 0 | - | - | - | - | - | - |
| *ZmG50* | GRMZM2G081671_T01 | intermediate | 0.00043 | 0 | 0.00022 | 0.40071 | 0 | 0.05909 | 0.03857 | 0.03868 | 9.74 | 5 | 0.083 |
| *ZmG51* | GRMZM2G333083_T01 | intermediate | 0.00063 | 0.00059 | 0.00063 | 0.43174 | 0.29129 | 0.01051 | 0.03041 | 0.03036 | 13.17 | 8 | 0.1061 |
| *ZmG53* | GRMZM2G026833_T01 | diversity | 0.00129 | 0.00064 | 0.00102 | 0.85018 | 0.66967 | 0.01098 | 0.0557 | 0.05569 | 18.392 | 12 | 0.1043 |
| *ZmG54* | GRMZM2G374986_T01 | diversity | 0.00112 | 0.00121 | 0.00119 | 0.8156 | 0.76877 | 0.00714 | 0.02005 | 0.02003 | 11.438 | 8 | 0.1781 |
| *ZmG55* | GRMZM2G124495_T01 | intermediate | 0.00038 | 0.00033 | 0.00037 | 0.40071 | 0.42943 | 0.00143 | 0.0211 | 0.02108 | 5.769 | 5 | 0.3294 |
| *ZmG56* | GRMZM2G016370_T01 | conserved | 0 | 0 | 0 | 0 | 0 | - | - | - | - | - | - |
| *ZmG57* | GRMZM2G159119_T01 | conserved | 0 | 0 | 0 | 0 | 0 | - | - | - | - | - | - |
| *ZmG58* | GRMZM2G090230_T01 | diversity | 0.00179 | 0.00222 | 0.00216 | 0.81649 | 0.78378 | 0.01043 | 0.07312 | 0.07302 | 21.358 | 16 | 0.1651 |
