# Supplementary material for: Molecular Evolution and Genetic Variation of G2-Like Transcription Factor Genes in Maize
Source: PLoS One. 2016 Aug 25;11(8):e0161763. doi: 10.1371/journal.pone.0161763 (PMC4999087; doi:10.1371/journal.pone.0161763)
Supplement: S7 Table — (DOCX) [file pone.0161763.s011.docx]

S7 Table qRT-PCR primers.

| Primer names | Primer sequence |
| --- | --- |
| ZmG1_F | GATTGATGGTTCCCAAAT |
| ZmG1_R | CCTTCCGTGTCATACTAAAA |
| ZmG2_F | TCATAGTAGGGAGATTTGTC |
| ZmG2_R | CAGGTCTGGTTTATAGCG |
| ZmG3_F | ACCGATTCTCACCTCTGC |
| ZmG3_R | AGGATTGTGAAGGCTAACG |
| ZmG11_F | AGCCTGTCCAGTCCTTTCTTT |
| ZmG11_R | CTTGAGGTGGTACAGCGTGAG |
| ZmG12_F | AAAGAACATAGGGAGCAAT |
| ZmG12_R | TTAAGTCTAGTAGGAGCGAGT |
| ZmG13_F | ACGCCTTGAGGCTTCCCGATACCTG |
| ZmG13_R | GTCGTCGTGTGGTGGTCCTCGT |
| ZmG25_F | CATCGTCGCTGGGAGTGGGT |
| ZmG25_R | TGGGTGTCGCCTTGTCTGC |
| ZmG26_F | GGCTGATGGGAATGAAA |
| ZmG26_R | TGGAATGCTAGGAGGAGGTAC |
| ZmG27_F | TCTTCTTCAGGCATGATGGG |
| ZmG27_R | ATGGTCTGGTAGGCTTTCTCC |
| ZmG28_F | TTGTCAAGGAGCATATCAG |
| ZmG28_R | GAGGCAGGAAAGGTAAAG |
| ZmG29_F | AGAGCAGGTGGAGTTTCA |
| ZmG29_R | CTCAGTCTTGGCTTGTTATT |
| ZmG34_F | TAGTTCTGTCGCCTCAAT |
| ZmG34_R | TCAACACTTGCTGGATAA |
| ZmG36_F | AGTAAAGCAGAGTTTGGGTA |
| ZmG36_R | ATTCAGCGACCTGTCTTG |
| ZmG38_F | TCATAATCGCTTTGTGGA |
| ZmG38_R | TGCATCTTTAGTGCCTCA |
| ZmG41_F | GACTCACCCTGTACCATCTCAA |
| ZmG41_R | CTCCCTTGGCATAGGGCGTTGTC |
| ZmG42_F | GCTGCCCAGGATATGAAA |
| ZmG42_R | GGAGATACTGAAGCCACTCG |
| ZmG43_F | CTGTTGGTCCTTTGTGCT |
| ZmG43_R | CTTGGGAAATCGGTTATG |
| ZmG44_F | TGGGAACAAGACCGATAA |
| ZmG44_R | CCAGCAGGAGTTTCAGATT |
| ZmG47_F | TCAACTGACGCTAAACCA |
| ZmG47_R | TTCTTGCTGAGCCTGTAT |
| ZmG50_F | AATGGATGGCTTCCTGACG |
| ZmG50_R | TTTGCCTGCTGCTCTTCG |
| ZmG55_F | GAGCAGCTCGAGATCCAGAGGAA |
| ZmG55_R | CAATTACAATGCCGACAAGCCAG |
